# Decoding the Genomic and Functional Landscape of Emerging Subtypes in Ovarian Cancer

**DOI:** 10.1158/2159-8290.CD-25-0652

**Published:** 2025-07-31

**Authors:** Giulia Micoli, Kari Lavikka, Yilin Li, Anna Pirttikoski, Daria Afenteva, Wojciech Senkowski, Giovanni Marchi, Anna Vähärautio, Taru A. Muranen, Titta Joutsiniemi, Sakari Hietanen, Anni Virtanen, Krister Wennerberg, Johanna Hynninen, Jaana Oikkonen, Sampsa Hautaniemi

**Affiliations:** 1 Research Program in Systems Oncology, Research Programs Unit, Faculty of Medicine, University of Helsinki, Helsinki, Finland.; 2 Biotech Research and Innovation Centre (BRIC), University of Copenhagen, Copenhagen, Denmark.; 3 Department of Obstetrics and Gynecology, University of Turku, and Turku University Hospital, Turku, Finland.; 4 Department of Pathology, University of Helsinki and HUS Diagnostic Center, Helsinki University Hospital, Helsinki, Finland.

## Abstract

Ovarian high-grade serous carcinoma (HGSC) is characterized by pervasive genomic instability and high inter- and intra-tumor heterogeneity. Approximately half of HGSC tumors harbor homologous recombination deficiency (HRD), rendering them vulnerable to PARP inhibitors and platinum-based chemotherapy. In contrast, patients lacking HRD (HR-proficient, HRP) generally respond poorly to current therapies. To overcome heterogeneity and identify relevant HGSC subtypes, we characterized the genomic landscape of 640 tumors from 243 patients using whole-genome sequencing. Our chromosomal instability signature–based analysis characterized the structural variation landscape and revealed five HGSC subtypes, validated in an independent dataset. Two HRD subtypes, associated with *BRCA1*- or *BRCA2*-driven alterations, demonstrated favorable treatment responses. Strikingly, three HRP subtypes emerged, marked by unique structural alterations and gene expression patterns, tumor microenvironment interactions, and different chemotherapy responses. Notably, organoid experiments showed subtype-specific sensitivity to CHK1 inhibition, suggesting prexasertib as a potential targeted treatment for most currently untreatable HRP patients.

**Significance:**

These findings demonstrate that HGSC tumors can be divided into functionally and clinically distinct subtypes, offering new insights into the underlying biology of HGSC and providing a foundation to develop tailored therapeutic strategies for HRP tumors, which currently lack effective options.

## Teaser

Five chromosomal instability–based ovarian cancer subtypes display distinct genomic, transcriptomic, and clinical features, with organoid studies revealing subtype-specific therapeutic sensitivities.

## Introduction

Ovarian high-grade serous carcinoma (HGSC) is the most abundant and aggressive form of ovarian cancer. Molecularly, it is characterized by chromosomal instability (CIN) and high intra- and inter-tumor heterogeneity (1–3). Currently, the only molecularly guided first-line treatment for HGSC targets tumors with *BRCA1/2* deficiency, or more broadly, homologous recombination deficiency (HRD), which are associated with greater sensitivity to poly (ADP-ribose) polymerase (PARP) inhibitors (4, 5). This is in sharp contrast with many other cancers, such as breast (6) and colorectal (7) cancers, in which molecular subtypes routinely guide treatment decisions. Critically, patients with HR-proficient (HRP) HGSC face significantly lower overall survival (OS) and have limited treatment options, underscoring the urgent need for more efficient therapies for HRP tumors (8).

In HGSC management, HRD status is commonly assessed using *BRCA1/2* mutation test or genomic scar assays, which detect specific patterns of genomic instability left in the genome due to defective DNA repair. These patterns include telomeric allelic imbalance, loss of heterozygosity, and large-scale state transitions (9). As a result, the proportion of patients with HRD HGSC ranges from 15% with *BRCA* mutations to approximately 50% with broader genomic approaches (10–12). Currently, HRP is defined only by the lack of HRD, and there are no established biomarkers. Whereas *CCNE1* amplification, *AKT2* amplification, and *CDK12* loss have been suggested to be enriched in HRP tumors (13), they do not capture the heterogeneous genomic landscape of these tumors.

In this study, we hypothesized that HGSC tumors can be stratified into distinct molecular subtypes with clinical relevance. With our approach, we leverage extensive structural variations that shape tumor heterogeneity and arise from pervasive genomic instability, which is an early event in HGSC evolution (14). Earlier studies have used copy number and structural variation signatures to investigate genomic complexity in HGSC. They identified copy number signatures as a key tool for understanding CIN (15), showed that multiple mutational processes shape HGSC genomes, making simple subtyping challenging (16), and suggested that HGSC genomes remain remarkably stable during chemotherapy (17). A significant limitation of these studies is their reliance on low-resolution data, such as shallow sequencing or array-based approaches, which may lack the precision needed to accurately capture the complex genomic landscape of highly heterogeneous HGSC tumors and the underlying mutational mechanisms driving the subtypes. Herein, we refine and extend the CIN signature analysis using whole-genome sequencing (WGS) data from more than 640 HGSC tumors collected in the real-world, observational, longitudinal DECIDER clinical trial, and discovered five robust HGSC subtypes. We characterized these subtypes with bulk and single-cell RNA sequencing (scRNA-seq) data. To validate the subtypes, we used 171 samples from 73 patients as an independent validation dataset. Importantly, we identified three distinct HRP subtypes and demonstrated, using organoid experiments, that the response to CHK1 inhibition varies significantly between these subtypes.

## Results

### Cohort and Extraction of CIN Signatures from WGS Data

**Figure 1.**
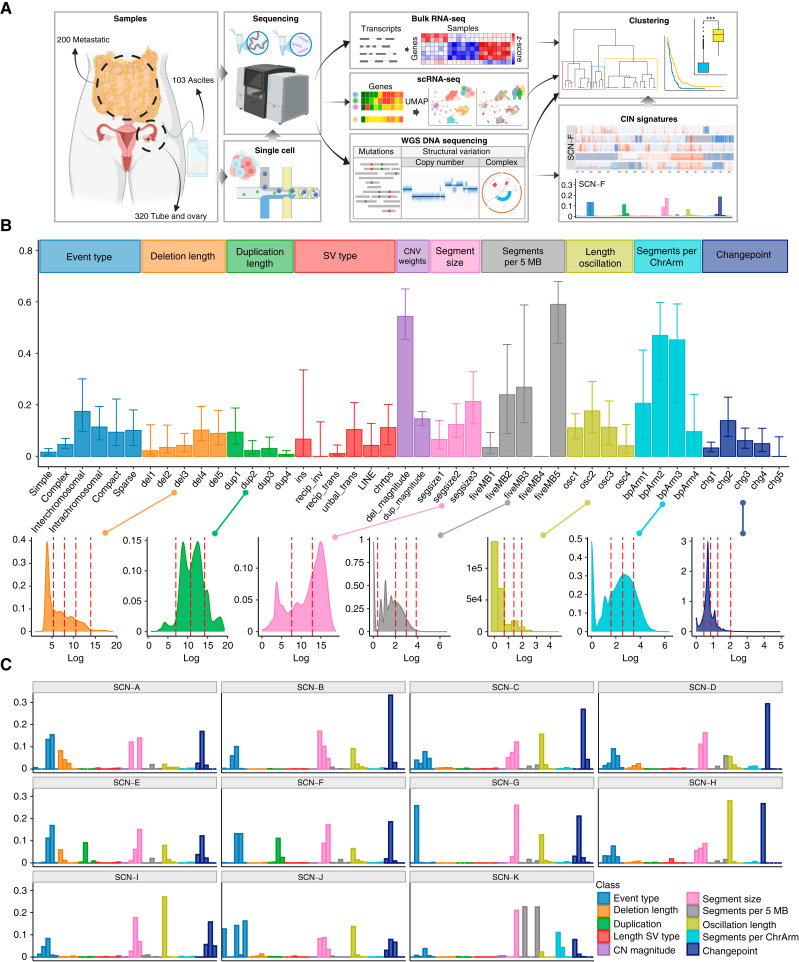
Study and signature overview. **A,** Schematic summary of the data and integration workflow. **B,** Feature summary: median of the value of each feature in the cohort, with error bars representing the quantiles 0.25 and 0.75. The features are colored according to the feature class and displayed in the boxes above the distribution. Feature classes derived from continuous features are connected below, with their distributions transformed on log scale. Colors match classes in A. The red dashed lines represent discretization thresholds determined using the Jenks natural breaks algorithm. CNV, copy number variant; LINE, long interspersed nuclear elements; SV, structural variant. **C,** Feature profiles of extracted signatures, derived with SigProfilerExtractor and using the features in A. The signatures exhibit distinct patterns, emphasizing their differences, and can be further observed in the interactive visualization at GenomeSpy_2.

Our discovery cohort consisted of 640 WGS samples from 243 patients, of whom 235 were diagnosed with HGSC. We included eight patients with other forms of ovarian cancer to assess the HGSC specificity of the results. All patients are enrolled in the longitudinal, multiregional, prospective, observational DECIDER trial (ClinicalTrials.gov ID: NCT04846933). The samples were obtained from different phases: before treatment (53%), after neoadjuvant chemotherapy (NACT) treatment (46%), and at relapse (7%). The analytic workflow is displayed in Fig. 1A, and a genomic snapshot of the cohort is shown in an interactive GenomeSpy (18) visualization at https://csbi.ltdk.helsinki.fi/pub/projects/micoli_et_al_2025/.

To discover robust CIN signatures, we first called somatic structural variation and copy number segments, as displayed in Supplementary Notes, and then quantified a comprehensive set of features for signature extraction. We expanded the existing copy number feature set (15) by incorporating novel structural variation features. As a result, we quantified 44 features from WGS data, grouped them into 10 broader categories, and analyzed their distributions (Table 1; Fig. 1B; Supplementary Notes).

**Table 1.** Graphical and textual explanation of the features used for the summarization of genomic profiles.

Feature	Graphical explanation	Description	Feature	Graphical explanation	Description
Simple event	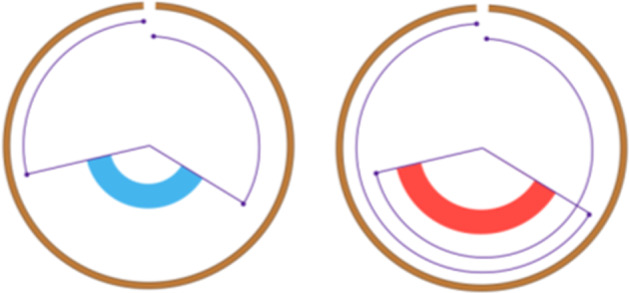	Single junction cluster which forms a local deletion, tandem duplication, or unbalanced translocation	Unbalanced translocation	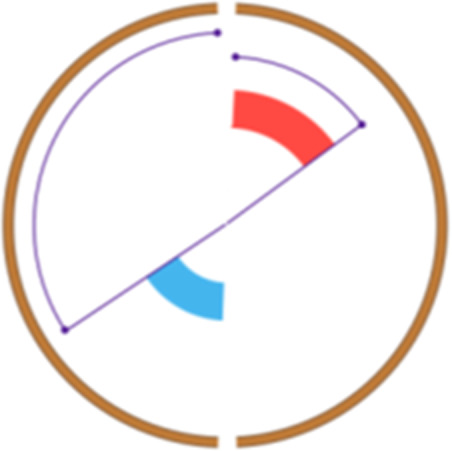	Uneven exchange of genetic material between chromosomes
Complex event	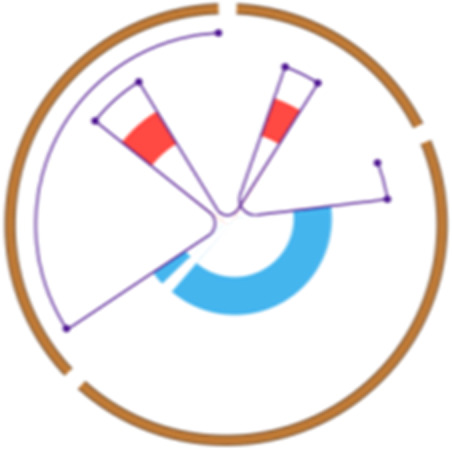	Clusters with three or more structural variants	Long interspersed nuclear element	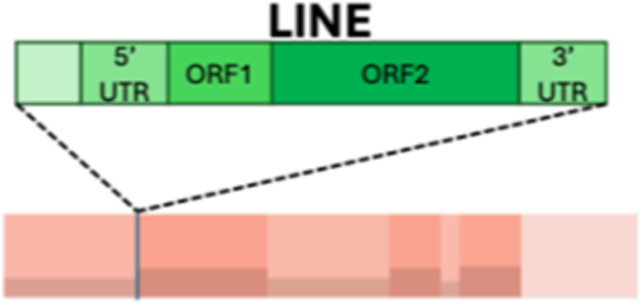	Insertion of long mobile DNA sequences and long interspersed nuclear elements
Intra/inter-chromosomal event	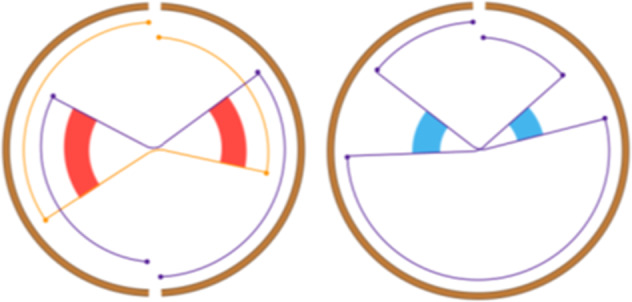	The junction cluster lies on one chromosome or is split into more chromosomes	Chromothripsis	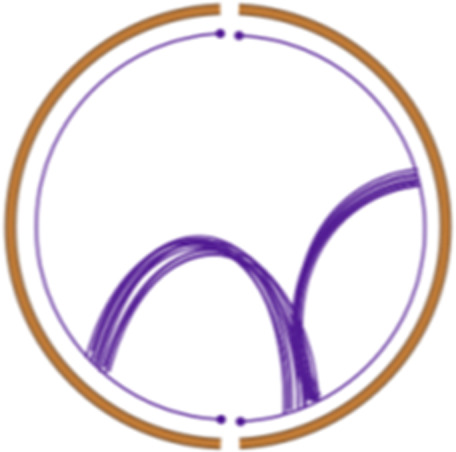	ShatterSeek evaluation of the high-confidence chromothripsis event (complex chromosomal rearrangement characterized by alternating copy number changes)
Compact event	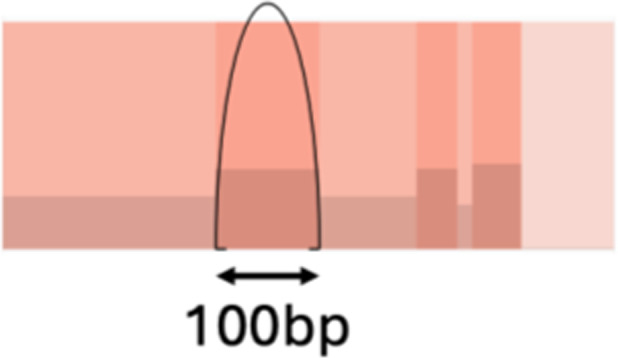	Breakpoints of the junction cluster are clustered in a restricted area of the chromosome	Deletion and duplication magnitude	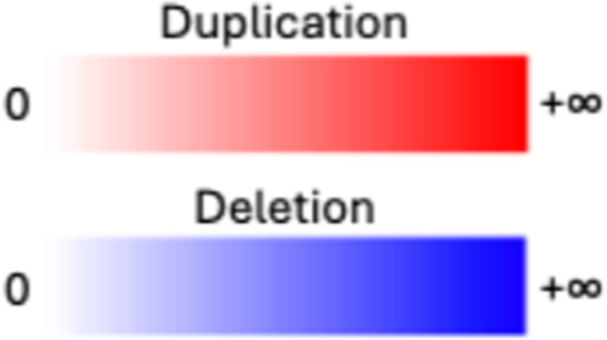	Quantiles 20^th^ and 80^th^ of the logarithm of the ratio between segment copy number and estimated sample ploidy. Measures how much the genome is deleted and amplified, respectively
Sparse event	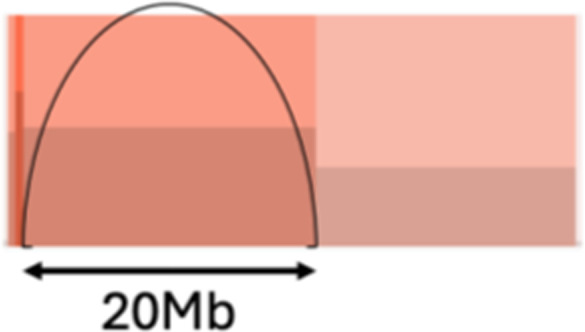	Breakpoints of the junction cluster are spread along the chromosome	Segment size	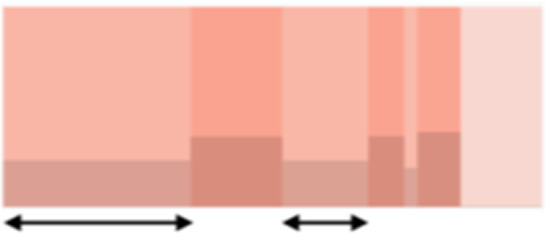	Length of the segments discretized in three categories using Jenks natural breaks
Deletion length	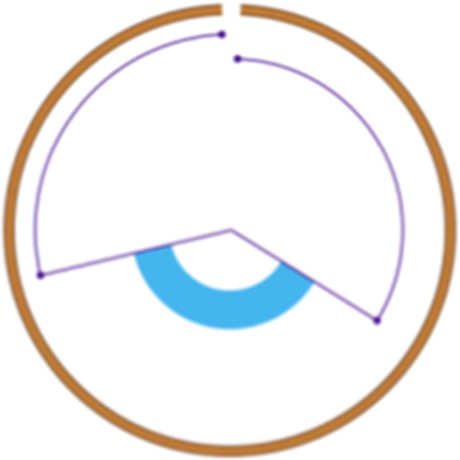	Number of clusters classified as deletions falling in discretized length ranges	Segments per 5 Mb	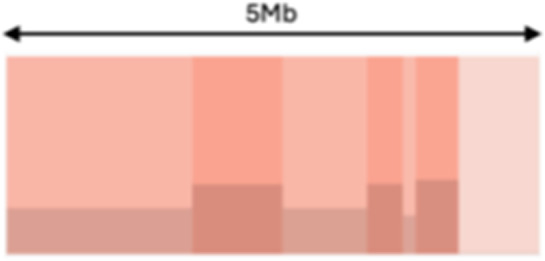	Number of segments in each 5 Mb window, discretized using mixture models
Duplication length	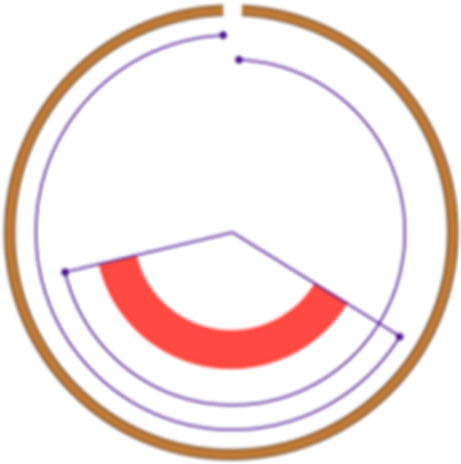	Number of clusters classified as duplications falling in discretized length ranges	Length oscillation chain	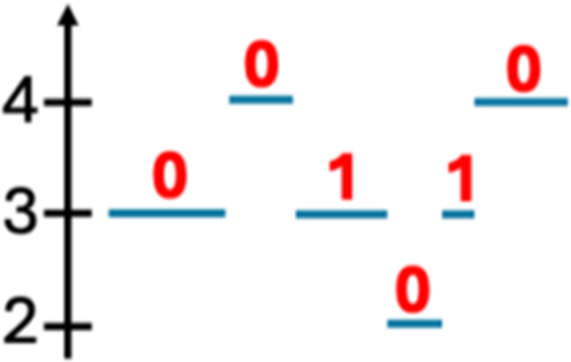	Length of segments chains characterized by and oscillating copy number, discretized with Jenks natural breaks
Insertion	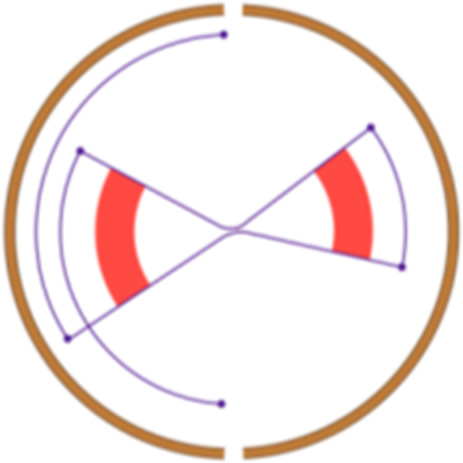	Number of events in which a segment of DNA is found in another region than the original	Segments per chromosome arm	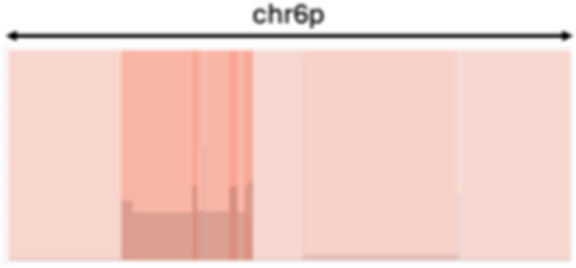	Number of segments per chromosome arm, discretized using Jenks natural breaks
Reciprocal	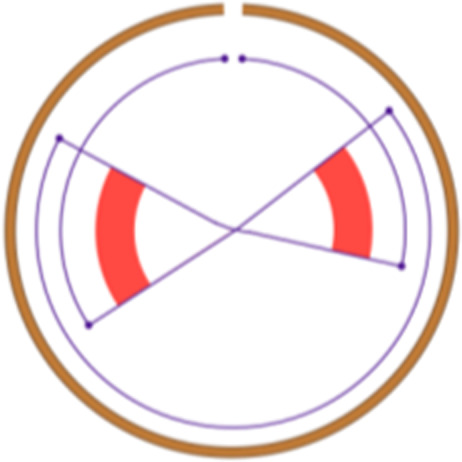	Reciprocal inversion or translocation events forming from two concurrent breaks interacting with each other	Changepoint	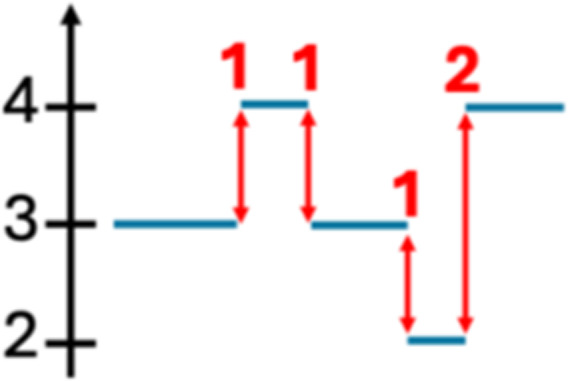	Difference in copy number with respect to the adjacent segments, discretized with Jenks natural breaks

We utilized the 44 features to extract *de novo* CIN signatures with SigProfilerExtractor (19), which resulted in 11 distinct CIN signatures that reveal unique patterns and distinguish different genomic landscapes in HGSC (Fig. 1C; Supplementary Notes). An overview of genome-wide patterns for these signatures is shown in GenomeSpy_2. To validate the robustness of the extracted signatures, we tested duplicated samples across different platforms, assessed signature consistency with varying SigProfilerExtractor parameters, and evaluated the impact of removing features (Supplementary Notes; Supplementary Fig. S1A–S1C). These tests show that the 11 CIN signatures are robust and enable comprehensive exploration of tumor genomes.

### Eleven Robust CIN Signatures Describe Distinct Biological Processes

**Figure 2.**
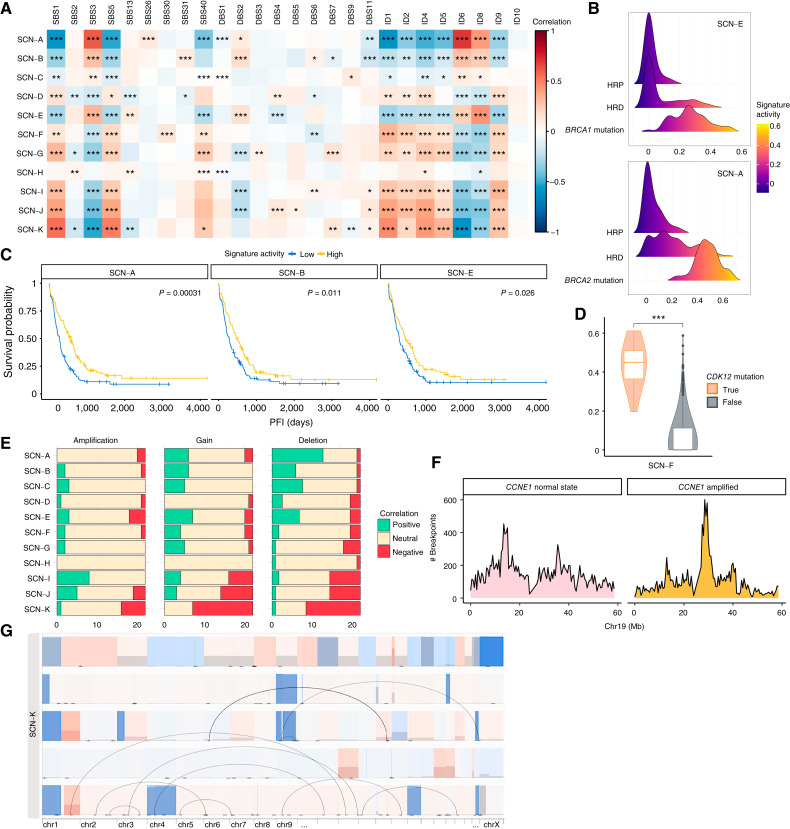
Biological and clinical associations of the SCN signatures. **A,** Correlation plot between COSMIC mutational and extracted signatures. Cell color indicates correlation strength and direction. Significance is assessed using Spearman’s correlation with FDR correction. **B,** Distribution of signature SCN-E (top) and SCN-A (bottom). Each panel displays the signature activity across HRP samples, HRD samples without the respective *BRCA* mutation (*BRCA1* for SCN-E and *BRCA2* for SCN-A), and samples with the corresponding *BRCA* mutation. Samples with *BRCA1* deficiency exhibit significantly higher SCN-E signature activity, whereas *BRCA2*-mutated samples show the same trend with SCN-A signature activity. Interactive visualization is available at GenomeSpy_3. **C,** Kaplan–Meier survival analysis of HRD-related signatures with log-rank *P*-values. Signature activities are first summarized per patient using the sample median. Patients are then divided into high and low signature activities using the median signature activity among all patients as a threshold. Elevated HRD-related signature activity is associated with improved treatment response. PFI, platinum-free interval. **D,** SCN-F signature activities for patients bearing *CDK12* mutation and patients not having it. Mann–Whitney U-test with FDR correction significance level is reported (*P* = 8.49e−10). **E,** Correlation between copy number alterations and signature activity. Bar plots display the number of amplifications (left), gains (middle), and deletions (right) derived from GISTIC, whose copy number–ploidy ratio correlates with the activity of each SCN signature. The length of each bar segment represents the number of copy number events in each correlation category. SCN-I and SCN-J display the highest number of positive correlations with amplified regions. Similarly, SCN-C exhibits a high number of positive correlations with gains and deletions, excluding HRD-related signatures. In contrast, SCN-K exhibits negative correlations with most deletion and gain peaks. **F,** Difference in breakpoints abundance in chromosome 19 between *CCNE1*amp samples and normal *CCNE1* samples. The counts are referred to bins of 0.5 Mb. **G,** GenomeSpy visualization of the signature SCN-K displaying the segmentation profiles of the five samples with the highest value for SCN-K activity, also available at GenomeSpy_5.

To enhance the interpretability of the extracted CIN signatures, we explored their associations with Catalogue of Somatic Mutations in Cancer (COSMIC) signatures, which represent known mutational processes across cancer types, as well as with HRD, major copy number events, and complex structural variations. First, we calculated the correlation between the existing COSMIC mutational signatures (20) and the 11 CIN signatures (Fig. 2A). Whereas most correlations were weak, some signatures showed stronger associations with HRD-related signatures. Signature SCN-A had the highest correlation with SBS3 (*r*_SCN-A_ = 0.60) and ID6 (*r*_SCN-A_ = 0.69). SCN-E was positively correlated with SBS3 (*r*_SCN-E_ = 0.35) and ID8 (*r*_SCN-E_ = 0.45). Additionally, SCN-B showed a correlation with SBS3 (*r*_SCN-B_ = 0.30) and ID6 (*r*_SCN-B_ = 0.30). Moreover, SCN-E and SCN-A were significantly enriched in *BRCA1*-mutated and *BRCA2*-mutated patients, respectively (Fig. 2B; Supplementary Fig. S2A–S2C; Genomespy_3), and their genomic characteristics aligned to *BRCA1/2* scars, i.e., genomic patterns marking mutations in these genes (21). Specifically, *BRCA1*-mutated tumors were enriched in the SCN-E signature, characterized by small deletions (0–200 bp) and duplications (1–45 kb), whereas *BRCA2*-mutated tumors exhibited numerous small to medium deletions (<36 kb) and a higher number of unbalanced translocations. Further supporting their link to HRD, signatures SCN-A, SCN-B, and SCN-E predicted mutational HR status (Supplementary Fig. S3A; Supplementary Notes) and were associated with favorable treatment response (Fig. 2C).

We then examined whether any of the 11 CIN signatures are associated with mutations beyond *BRCA1/2*, specifically *CDK12* and *NF1*, as well as the most frequently altered copy number regions. The signature SCN-F was significantly enriched in tumors with *CDK12* mutations (Fig. 2D), whose prevalence is 4% in the DECIDER cohort, and its features align with the tandem-duplicator phenotype (22). SCN-A was found to be enriched in tumors with losses and deletions in the *NF1* gene, which regulates the RAS-MAPK signaling pathway, and is dysfunctional in 20% of cases in the DECIDER cohort (Supplementary Fig. S3B). To study the link between signatures and copy number changes, we utilized Genomic Identification of Significant Targets in Cancer (GISTIC) to identify amplification, gain, and deletion peaks (Supplementary Notes). GISTIC is a computational method used to identify genomic regions that are significantly and recurrently amplified or deleted across a cohort (23). Apart from the HRD-related signatures, SCN-C was associated with most gains and deletions, suggesting extensive genome alterations, whereas SCN-I and SCN-J were mainly linked to amplifications (Fig. 2E). Further analysis of these three signatures revealed their association with tumors that underwent whole-genome duplication and the recurrently amplified HGSC genes *CCNE1*, *KRAS*, *MYC*, and *MECOM* (Supplementary Fig. S3C–S3E; ref. 2). Additionally, high activity of SCN-D, SCN-I, and SCN-J signatures was indicative of poorer treatment response (Supplementary Fig. S3F).

Next, we investigated the association of the signatures with previously described structural variation classes (24). Tyfonas, clusters of copy number segments with fold-back inversions resembling a typhoon, were primarily linked to SCN-I. Breakage–fusion–bridge (BFB) cycles, which involve chromosome breakage, faulty fusion, and repeated rearrangements, were associated with SCN-I and SCN-J. SCN-F was linked to pyrgos, characterized by stacked amplified copy number segments, whereas SCN-B was associated with chromoplexy, in which multiple chromosomes break and reconnect incorrectly (Supplementary Fig. S4A–S4H). Interestingly, BFB cycles in SCN-I and SCN-J mainly occurred on chromosome 19, including *CCNE1* (Supplementary Fig. S4H; GenomeSpy_4), and breakpoints on chromosome 19 peaked only in *CCNE1*-amplified samples (Fig. 2F), suggesting that BFB cycles drive *CCNE1* amplification.

Lastly, SCN-K exhibited a pattern with few long segments, near-normal ploidy, and minimal structural variants, suggesting a reference genome state. We define such patterns as simple genomes (Fig. 2G; GenomeSpy_5). In contrast, SCN-G, predominantly found in low-purity (∼0.20) ascites samples, showed recurrent deletions unsupported by structural variation calls, likely artifacts confirmed by their absence at the read level (Supplementary Fig. S5; GenomeSpy_5). These findings highlight the diverse genomic landscapes captured by the 11 CIN signatures, which are summarized along with their molecular associations in Table 2.

**Table 2.** Summary of biological associations and characteristics attributed to each signature.

Signature	Drivers and complex structural variants	Mutational signatures	Whole-genome duplication	Genomic characteristics	Explanation
SCN-A	*BRCA2* mutation	SBS3, ID6, and ovaHRDscar	No	Small deletions, reciprocal and unbalanced translocations, simple structural variants, and low changepoint	*BRCA2* mutation–driven HRD, clinically significant
SCN-B	General HRD and chromoplexy	SBS3, ID6, and ovaHRDscar	No	Interchromosomal events (chromoplexy), short segment size, short oscillation chains, and low changepoint	Unknown HRD mechanisms, clinically significant
SCN-C	GISTIC gains, *MYC* amplification, and rigma		Yes, high ploidy	Short–medium segment size, high deletion and duplication magnitude, high number of breakpoints per 5 Mb, and extensive loss of heterozygosity	Whole-genome duplication followed by high rearrangement of the genome, *MYC*-driven pattern
SCN-D				Sparse events, long segments interspersed with short ones	
SCN-E	*BRCA1* mutation	SBS3 and ID8	No	Highly fragmented, simple structural variants, short deletions and short duplications, short to medium segment size, and reciprocal translocations	*BRCA1* mutation–driven HRD, clinically significant
SCN-F	*CDK12* mutation and pyrgo			Short to medium duplications, highly fragmented, medium to high length of oscillation chain, and high breakpoints per chromosomal arm	*CDK12* mutation–driven pattern of tandem duplications and pyrgo
SCN-G				Low purity and high number of complex events	Segmentation model aberration
SCN-H				Short oscillation chains, low changepoint, and no high number of long interspersed nuclear element events	
SCN-I	GISTIC amplification, *CCNE1* amplification, tyfonas, BFB, and double minutes		Yes, high ploidy	High changepoint, medium to long segment sizes, low number of breakpoints, and high number of interchromosomal events	Whole-genome duplication followed by tyfonas and BFB events *CCNE1* amplification–driven pattern
SCN-J	GISTIC amplification, *CCNE1* amplification, and BFB		Yes. high ploidy	High number of sparse events, all segment sizes, low to medium number of breakpoints per 5 Mb, and chromosomal arm	Whole-genome duplication followed by BFB events, *CCNE1* amplification–driven pattern
SCN-K		SBS1 and SBS5		Almost absent structural variants, low number of breakpoints per 5 Mb, and chromosomal arm	Marker of simple genomes, patients without HGSC

### CIN Signatures Reveal Two HRD and Three HRP Subtypes in HGSC

**Figure 3.**
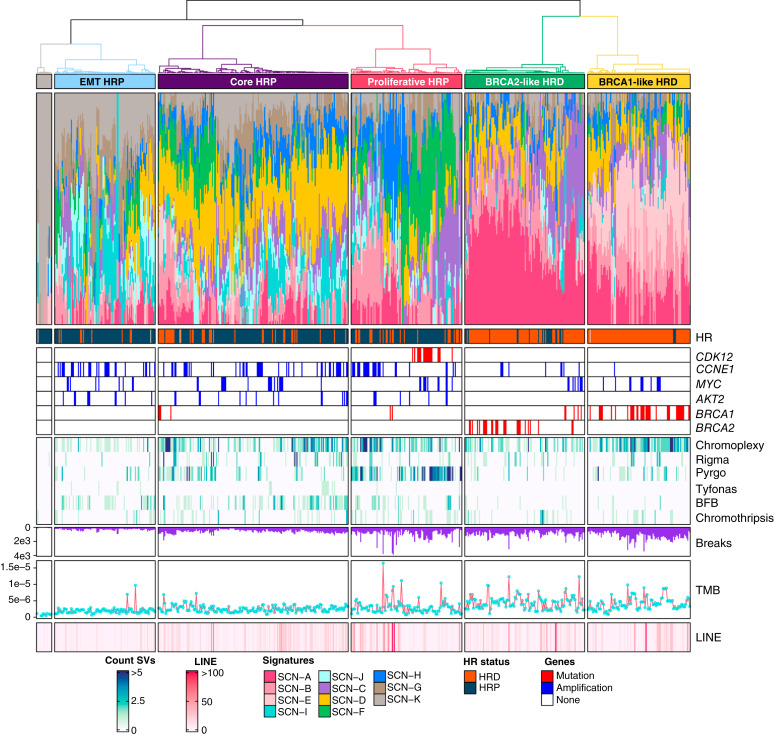
Subtype discovery. The SCN signature activity profiles have been clustered into six subtypes with ConsensusClusterPlus and annotated in different colors beneath the dendrogram. The accompanying barplot illustrates signature activity values, with each color representing a distinct signature. Annotations below the main barplot include mutational HR status and the presence of specific gene alterations, such as *CDK12*, *BRCA1*, and *BRCA2* mutations, as well as *CCNE1*, *MYC*, and *AKT2* amplifications. A heatmap also displays the counts of complex structural variations (SV) using a color-coded scale. The purple bar plot below indicates the number of breakpoints, the red track with light blue dots represents the tumor mutational burden (TMB), and the amount of long interspersed nuclear elements (LINE) in each sample is shown as a heatmap in the bottom annotation. Notably, *AKT2* amplification and *CDK12* mutation are mutually exclusive to HRD clusters, whereas *CCNE1* and *MYC* amplifications are present in both HRP and HRD compartments. Moreover, HRD subtypes and the proliferative HRD subtype are characterized by high TMB and number of breaks, and the proliferative subtype is enriched in pyrgos and chromoplexy. Further visualization of the clusters is available at GenomeSpy_6.

**Figure 4.**
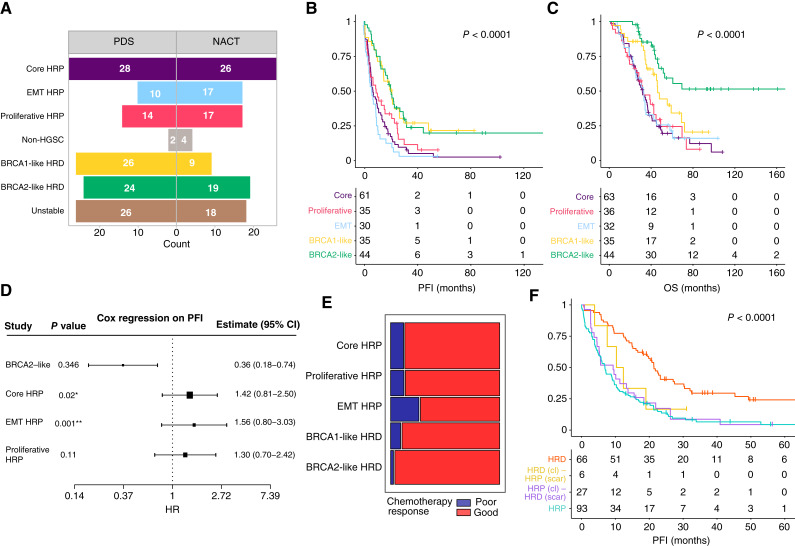
Clinical relevance of the subtypes. **A,** Distribution of patients across molecular subtypes stratified by treatment strategy: primary debulking surgery (PDS) and NACT. Unstable patients are those whose samples fall into multiple clusters. **B,** Kaplan–Meier survival analysis of platinum-free interval (PFI) across the subtypes (*P* < 0.0001). Unstable patients have been excluded. Patients in BRCA1-like and BRCA2-like subtypes show improved response to chemotherapy. **C,** Kaplan–Meier survival analysis of OS across molecular subtypes (*P* < 0.0001). Exclusion criteria like in B. BRCA2-like HRD cluster shows the most favorable prognosis. **D,** Cox proportional hazards model assessing the association between subtypes and PFI. BRCA1-like HRD was used as the reference category. The EMT HRP cluster was significantly associated with worse outcomes. The overall model was significant (log-rank *p* = 2e−5) with a concordance index of 0.635, indicating good discriminative ability. **E,** Primary chemotherapy response across molecular subtypes. The width of each bar is proportional to the number of patients in the respective subtype, reflecting the sample size distribution across groups. Poor responders are predominantly found in the HRP subtypes, with the EMT HRP subtype having the highest proportion. **F,** Kaplan–Meier survival analysis of 5 years OS comparing HR status determined by ovaHRDscar and molecular clustering (*P* < 0.0001). Cluster-defined HRD patients include stable patients within BRCA1-like and BRCA2-like subtypes. Molecular clustering identifies a subgroup of HRP scar patients in which 65% survived beyond 3 years after diagnosis (purple), compared with only 32% survival among patients classified as HRP by both methods (light blue).

To identify patient subtypes, we clustered iteratively 629 selected HGSC samples based on their CIN signature activity (Supplementary Notes). This analysis resulted in six clusters characterized by combinations of the 11 CIN signatures (Fig. 3; GenomeSpy_6). Stability analysis of the clusters showed that approximately 81% of patients had all their samples assigned to the same cluster. Briefly, we identified two HRD subtypes—BRCA1-like (80% *BRCA1*-mutated samples), BRCA2-like (all *BRCA2*-mutated and *RAD51c/d*-mutated samples)—and three HRP subtypes. Non-HGSC patients, specifically all patients with low-grade ovarian cancer, and HGSC with simple genomes showed high SCN-K activity, forming a non-HGSC cluster. In total, approximately 35% of stable patients belong to the HRD subtypes. Treatment strategies were generally evenly distributed across subtypes, except for the BRCA1-like HRD subtype, which consisted of the majority of primary debulking surgery cases, and the epithelial–mesenchymal transition (EMT) subtype, which was enriched with patients who underwent NACT (Fig. 4A).

To evaluate the clinical significance of the identified subtypes, we analyzed their association with patient outcomes and treatment responses. Analysis of platinum-free interval and OS across molecular subtypes revealed distinct clinical differences in prognosis. As expected, BRCA1-like and BRCA2-like HRD subtypes had better outcomes with standard treatment than the HRP subtypes (Fig. 4B), reflecting their sensitivity to platinum-based chemotherapy and PARP inhibitors. Patients with BRCA2-like tumors exhibited significantly longer OS compared with any other subtype (Fig. 4C), indicating sustained platinum sensitivity and a durable PARP inhibitor response. Importantly, as these subtypes also include patients without *BRCA1/2-*mutated tumors, this classification effectively identifies patients who are likely to benefit from chemotherapy and possibly PARP inhibitors.

A Cox regression model further revealed that the core HRP and EMT HRP subtypes were significantly associated with worse platinum-free interval, presenting clinical differences between HRP subtypes. Moreover, the EMT HRP subtype showed the highest risk (Fig. 4D). Next, we evaluated chemosensitivity after surgery and compared primary chemoresponse across subtypes. Patients with poor or no response to the first-line therapy were most common in the EMT HRP subtype (Fig. 4E). These results suggest an intrinsic resistance in the EMT subtype related to its specific genomic landscape.

To assess whether the subtype associations could be reproduced in an independent dataset, we analyzed 171 tumor samples from 73 patients with HGSC. Using a random forest model (Supplementary Notes), we quantified CIN signatures and classified the samples into the six subtypes. The distribution of subtypes mirrored that of the discovery cohort (Supplementary Fig. S6A), with 80% of patients showing stable subtype assignments. Genomic features of the subtypes in the validation dataset were consistent with those observed in the discovery cohort (Supplementary Fig. S6B). Furthermore, a multivariate survival meta-analysis revealed no significant heterogeneity between the two cohorts (I^2^ = 1%, Supplementary Fig. S6C and S6D).

Next, to assess the clinical value of our clustering approach, we compared its ability to detect HRD patients against genomic scar test (11). The CIN-based subtypes demonstrated predictive accuracy comparable with that of ovaHRDscar, with a concordance probability estimate of 0.60 for the CIN-based HRD classification and 0.58 for ovaHRDscar. To further compare the two HRD detection methods, we investigated the overlap between their classifications. Six patients were classified as HRD based on the CIN signature subtypes but HRP by scar, and 27 were classified as HRP based on the CIN signature subtypes but HRD by scar. Kaplan–Meier survival analysis shows that among patients classified as HRD by scar test but assigned to HRP subtypes, 93% relapsed within 3 years after diagnosis, compared with only 78% of those identified as HRD by both methods (Fig. 4F). This suggests that CIN-based HRD/HRP classification may provide a more precise approach than traditional genomic scar analysis.

### Molecular Characterization of the HRP Subtypes

**Figure 5.**
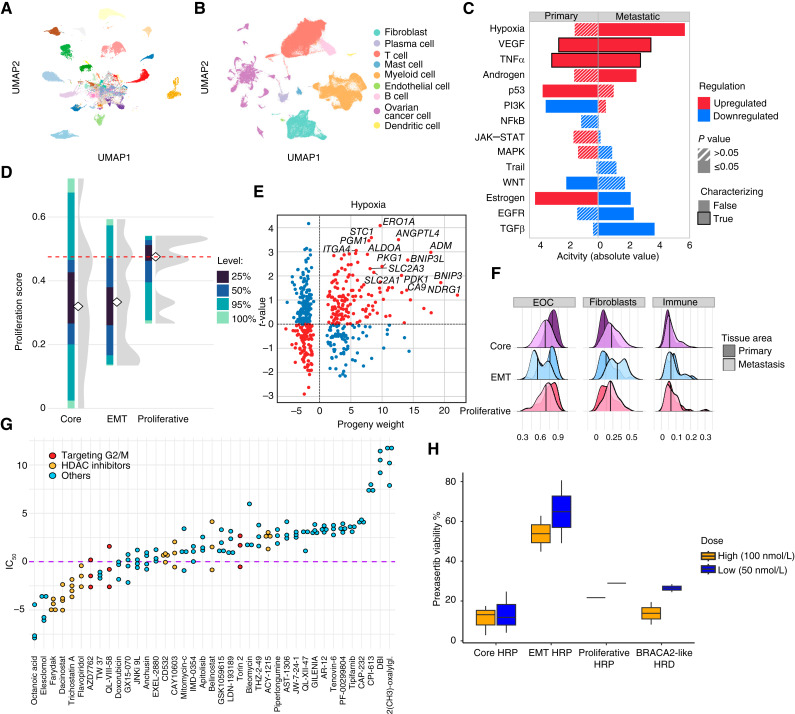
Subtype characterization. **A,** Uniform Manifold Approximation and Projection (UMAP) of scRNA-seq cancer cell profiles from the full dataset of 57 patients, colored by patient. **B,** UMAP of scRNA-seq profiles from the 57 patients colored by cell type. **C,** Pathway activities of proliferative subtype samples from analysis of the primary and metastatic tissues. The bar color represents the regulation direction of the pathway, the white stripes pattern indicates nonsignificant results, and the stroke highlights the characterizing pathways (pathways that exhibit the same directional trend in both tissue compartments). **D,** Proliferation score distribution across the core, EMT, and proliferative subtypes, calculated as the proportion of cells in S and G2/M cell-cycle phases per scRNA sample. Colors represent quantiles, white diamonds indicate median scores, and the red dashed line marks the proliferative HRP reference. Gray densities show the overall score distribution within each subtype. **E,** Progeny-derived weights and t-values from differential expression analysis (DEA) of hypoxia target genes in metastatic samples of the proliferative subtype. Red dots represent significantly upregulated genes, whereas blue dots indicate downregulated genes. **F,** Distribution of epithelial ovarian cancer (EOC), fibroblast, and immune cell counts across tissue areas (primary and metastatic) for the HRP subtypes. Notably, metastatic EMT subtype samples display a significantly higher proportion of fibroblasts than the other HRP subtype (Mann–Whitney U test, *P* = 0.02). **G,** IC_50_ values for HRP cell lines from the GDSC1 drug screen (ES2, NIHOVCAR3, FUOV1, JHOS2, and OV90). Compounds targeting the G2/M cell-cycle checkpoint and histone deacetylase (HDAC) inhibitors are highlighted. HRP cells exhibit sensitivity to both classes of compounds, suggesting a potential vulnerability associated with G2/M checkpoint regulation. **H,** Viability of patient-derived organoids (PDOs) after prexasertib delivery in two concentrations. Notably, core HRP and BRCA2-like HRD subtypes are sensitive, whereas EMT HRP is resistant.

We analyzed the genomic features of HRP subtypes (GenomeSpy_7) and their transcriptomic differences based on transcription factor (TF) and pathway activities. Given the known differences between site-of-origin (fallopian tube and ovary) and metastatic (omentum, peritoneum, and bowel mesentery) tissues, we compared site-of-origin and metastatic samples separately across and within the groups (site-of-origin vs. metastatic). We also assessed cellular composition and cancer cell proliferation using scRNA-seq (Fig. 5A and B) and examined their association with evolutionary states (3).

### Proliferative HRP Subtype

The subtype characterized by signatures SCN-F, SCN-B, and SCN-C had the highest number of breakpoints, chromoplexy, and pyrgos among the HRP groups (Supplementary Fig. S7A and S7B). It also had a high prevalence of *CSMD3* mutations (29% of patients), which are associated with high tumor mutational burden (TMB) and immune infiltration (25). Other key mutations included *CDK12* (26%), *LAMA* (16%), and *USH2A* (13%).

In both site-of-origin and metastatic tissues, TF analysis (Supplementary Notes) showed increased *NFKB* and *AR* activity. In addition, pathway analysis revealed higher androgen signaling and suppressed estrogen signaling in the metastatic compartment (Fig. 5C; Supplementary Fig. S7C–S7F). ScRNA-seq confirmed a higher proliferation score than other HRP subtypes (*P* = 0.03; Fig. 5D), supported by two proliferation signatures (Supplementary Notes; refs. 26, 27). Moreover, cell type analysis revealed an increased abundance of M2 macrophages (Supplementary Fig. S7G). Altogether, these findings suggest a proliferative phenotype for this HRP subgroup.

In metastatic tumors, pathway and TF analyses revealed strong hypoxia-inducible factor-1α–driven hypoxia activation, influencing glycolysis (*SLC2A1*, *SLC2A3*, *PGK1*, *PDK1*, *PGM1*, and *ALDOA*), angiogenesis (*ANGPTL4* and *ADM*), and survival in low oxygen (*BNIP3*, *BNIP3L*, and *NDRG1*; Fig. 5E; Supplementary Fig. S7H). This was further validated by Buffa hypoxia signature (Supplementary Notes; ref. 28). Together, these findings highlight the key roles of proliferation and hypoxia in this subtype.

### EMT HRP Subtype

The subtype defined by SCN-I and SCN-J had the lowest number of breaks and structural variations and the lowest TMB among HRP subtypes. It showed the highest mutation rates in *RB1* (14%) and a copy number landscape with few segments. The only consistent hotspot was located on chromosome 19 and associated with *CCNE1* amplification.

Both site-of-origin and metastatic samples showed low levels of hypoxia, reflected by low *HIF1a* and *EPAS1* activities (Supplementary Fig. S8A–S8D). The metastatic compartment exhibited high activity of *FOXF2* transcription factor and EGFR and TGF-β pathway activities, suggesting an invasive phenotype driven by EMT (Supplementary Fig. S8D–S8F). Indeed, scRNA-seq data revealed a prevalence of B cells (*P* = 0.005) and mast cells (*P* = 0.02), which are known EMT inducers (29). Pathway analysis supported the involvement of EMT in this subtype (Supplementary Fig. S8G). Additionally, cell type bulk RNA analysis showed fewer cancer cells and more fibroblasts than the other HRP subtypes in metastatic samples (Fig. 5F). This is consistent with scRNA-seq data that showed the highest fibroblasts/tumor ratio among all HRP subtypes (*P* = 0.02, Supplementary Fig. S8H), a feature strongly linked to EMT. Overall, this HRP subtype is characterized by low hypoxia and high EMT.

### Core HRP Subtype

The samples characterized by SCN-D and SCN-H exhibited a significantly higher number of long interspersed nuclear element events (Supplementary Fig. S9A) and intermediate levels of structural variation, breaks, and TMB compared with the other HRP subtypes. Only a few genes—*CCDC168*, *FAT3*, *FLG*, *OTOG*, and *SCN2A*—were mutated in more than 10% of patients.

Transcriptomic analysis of site-of-origin samples showed high hypoxia activity, low JAK-STAT signaling, and downregulation of RFX complex components, potentially reducing MHC-II expression and promoting immune evasion (Supplementary Fig. S9B and S9C; ref. 30). scRNA-seq revealed the lowest number of fibroblasts among HRP subtypes and a significant abundance of immune-evasive myeloid cells (*P* = 0.03).

Pathways and TF activities in metastases differed from site-of-origin tumors, with hypoxia and JAK-STAT showing opposite activity changes—hypoxia increasing whereas JAK-STAT decreasing (Supplementary Fig. S9D–S9F)—suggesting a significant reprogramming of transcriptional regulation. Only a few genes were differentially expressed compared with the other HRP subtypes (Supplementary Fig. S9G), indicating extensive transcriptional heterogeneity within this group and thus a less separate molecular profile. We also observed a higher prevalence of samples characterized by the adaptive and maintaining evolutionary states, denoting increased heterogeneity in metastatic samples and high intrasample heterogeneity (Supplementary Fig. S9H; ref. 3). Taken together, in the core HRP subtype, site-of-origin tumors exhibit immune evasion through RFX complex suppression and myeloid cell infiltration, whereas metastases are more heterogeneous and do not consistently display immune evasion features.

### Core HRP Tumors Are Sensitive to CHK1 Inhibitor Prexasertib

To suggest targeted therapies for the HRP subtypes, we first utilized existing *in vitro* drug experiment repositories (31, 32), followed by validation with organoids. We used genomics data to identify cell lines belonging to the HRP subtypes and identified five HRP cell lines. We then screened for compounds that induced cell death as monotherapies and exhibited differential effects across these lines. Several histone deacetylase inhibitors and two compounds targeting the G2/M cell-cycle checkpoint emerged as broadly effective (Fig. 5G). The drugs that showed the strongest cell line-specific responses were the CHK1 inhibitor AZD7762, the ATR inhibitor QL-VIII-58, and the histone deacetylase inhibitor belinostat.

As CHK1 is a key driver in the G2/M cell-cycle checkpoint pathway, which differed between the HRP subtypes in the RNA-seq data, we evaluated the efficacy of CHK1 inhibition using patient-derived organoids (PDOs) from the DECIDER trial. PDOs were treated with two doses of prexasertib, which is an investigational CHK1 inhibitor. Core HRP subtype PDOs exhibited significantly higher sensitivity to prexasertib compared with the EMT HRP subtype PDOs (*P* = 0.005, Fig. 5H). Additionally, the proliferative HRP and BRCA2-like subtype PDOs demonstrated sensitivity to prexasertib, although their response was less pronounced than that of core HRP PDOs. These results provide preclinical evidence for the utility of CHK1 inhibitors as a targeted therapy for a molecularly defined subset of patients with HRP tumors.

## Discussion

We characterized the HGSC genomic landscape using CIN signatures based on patient WGS data, which enabled the discovery of clinically relevant HGSC subtypes. These subtypes exhibit distinct structural variation profiles reflecting different mutational processes and biological phenotypes. Importantly, three subtypes further stratified the currently poorly responding and heterogeneous patient group with HRP tumors. Furthermore, we identified signatures that allowed us to identify non-HGSC samples based on their genomic features.

The largest HRP subtype, core HRP, consists of 48% of patients with HRP tumors. It displayed medium genomic instability and structural variation with a predominantly immune-desert microenvironment. Signs of immune evasion were most evident in fallopian tubes and ovaries, whereas metastases shifted toward hypoxia and revealed high heterogeneity, suggesting phenotypic plasticity. In contrast, the EMT HRP subtype accounts for 25% of HRP tumors and exhibits the lowest genomic instability among HRP subtypes, with minimal structural variation and a simple copy number landscape, except for recurrent *CCNE1* amplification. Tumors in the EMT HRP subtype displayed a fibroblast-rich microenvironment, low hypoxia and proliferation, and strong EMT-driven invasiveness, supported by *FOXF2*, EGFR, and TGF-β activity, as well as increased mast cell and B-cell infiltration. The proliferative HRP subtype (27% of HRP patients) exhibited the highest genomic instability, characterized by elevated breakpoints, chromoplexy, and genome doubling. Tumors in this subtype displayed a proliferation-driven phenotype, characterized by androgen signaling and an M2 macrophage-enriched microenvironment, whereas metastases showed strong hypoxia activation. Notably, *CSMD3* mutations and *CDK12* loss were identified in 52% of proliferative HRP patients. Both genes are known to drive high TMB (25, 33), suggesting their role as key drivers of the proliferative subtype. Furthermore, *CDK12* loss–related genomic patterns have been detected with other genomic signature frameworks (16, 34), emphasizing the importance of *CDK12* in cancer progression.

The previously proposed HRP markers, *CDK12* loss and *AKT2* amplification, were found exclusively in HRP tumors. *CCNE1* amplifications were enriched in HRP tumors but also observed in HRD tumors. This is in line with earlier studies in which *CCNE1* amplifications were also found in long-term HGSC survivors, whose tumors are typically HRD (35). Other driver differences between HRP subtypes, such as *RB1* loss, primarily seen in EMT, and *CSDM3* mutation in the proliferative HRP subtype, are likewise detected in HRD patients.

As expected, patients across all HRP subtypes exhibited poor responses to standard-of-care treatment. Encouragingly, our organoid data showed distinct subtype-specific responses to CHK1 inhibition: core HRP tumors showed potential sensitivity to prexasertib, EMT HRP tumors demonstrated no response, and proliferative HRP organoids exhibited moderate sensitivity. These results suggest that the EMT subtype could be used as a contraindication for prexasertib in HGSC, whereas patients with proliferative and especially core HRP subtypes should benefit from this treatment. Prexasertib has shown single-agent activity in a subset of patients with recurrent HGSC in a phase II trial, but no link to specific genomic alterations was found (36). Recently, Yang and colleagues identified synthetic lethal vulnerabilities associated with *CDK12* loss and demonstrated the efficacy of CHK1 inhibition (37). These findings align with our results for the proliferative HRP subtype, which is enriched in tumors harboring *CDK12* loss, further supporting the potential of CHK1 inhibition for patients with proliferative and core HRP subtypes.

Among HRP subtypes, chemotherapy response differs markedly. In particular, the EMT subtype carries a higher risk of recurrence compared with the proliferative subtype. This reflects growing evidence that EMT promotes chemotherapy resistance, particularly in HGSC. EMT-associated tumors are often characterized by low tumor purity and extensive fibroblast infiltration—features of a stroma-rich microenvironment that fosters drug resistance and immune evasion (38, 39). In contrast, the proliferative subtype, defined by high mitotic activity, tends to respond more effectively to chemotherapy, likely due to its intrinsic sensitivity to DNA-damaging therapies. The lack of response in the EMT subtype is consistent with its reduced proliferative activity, making it less susceptible to cell cycle–targeting agents, such as prexasertib.

Previous approaches using lower-resolution data were unable to distinguish *BRCA1*- from *BRCA2*-mutated tumors and define biologically meaningful HRD and HRP subtypes (15, 16, 34, 40). Furthermore, our results suggest that scar-based tests, such as ovaHRDscar, may overestimate HRD status. Our approach, combined with high-resolution WGS data, enabled the separation of patients with dysfunctional *BRCA1* and *BRCA2* genes. Patients in the BRCA1-like subtype responded to standard-of-care treatment similarly to those in the BRCA2-like subtype and had similar times to progression. However, BRCA2-like patients exhibited better OS, consistent with previous studies (41, 42), suggesting better responses and a lower rate of resistance to second-line treatments compared with BRCA1-like patients. Notably, clinical characteristics, such as age, Federation Internationale des Gynaecologistes et Obstetristes (FIGO) stage, or body mass index, did not account for the observed differences in survival between the two subtypes. This difference may reflect the distinct roles of *BRCA1* and *BRCA2* in HR. It has been proposed that BRCA2, being more centrally involved in the HR repair process, may confer greater sensitivity to treatment when lost, thus potentially leading to improved outcomes (41–43).

In conclusion, we have characterized the genomic landscape of HGSC tumors in a real-world patient cohort and identified biologically and clinically relevant subtypes through CIN signatures. The subtypes were validated in an independent dataset. The herein discovered subtypes provide a foundation for developing new therapeutic strategies for patients with currently untreatable HGSC.

### Limitations

We were not able to identify a distinguishing biological explanation for 2 of 11 signatures. These two signatures were enriched in the core HRP subtype and could highlight novel biological mechanisms. Differences within the three HRP subtypes suggest the need for further refinement, such as the characterization of subgroups enriched for *CDK12* loss or *CCNE1* amplification within the proliferative HRP subtype. However, identifying these finer subtypes would require larger, dedicated cohorts. We were able to propose a potentially efficient treatment option for patients with tumors in the core and proliferative HRP subtypes but not for the EMT HRP subtype. Our work, however, provides stratification of the HRP tumors into distinct subtypes, which should facilitate more personalized treatment options. It is also worth noting that the cohort is primarily composed of genetically similar patients, which may limit the generalizability of the findings. Validation in more genetically diverse populations will be an important direction for future studies.

## Methods

### Cohort Description

The study involved a total of 316 female patients diagnosed with ovarian cancer, divided into a discovery cohort (*n* = 243) and a validation cohort (*n* = 73). Eight patients in the discovery cohort were diagnosed with another histologic subtype (four low-grade serous carcinoma, two endometrioid, one carcinosarcoma, and one mesonephric-like epithelial), and all other patients were diagnosed with HGSC, verified by a pathologist. All patients were enrolled in DECIDER, a prospective, longitudinal, multiregion observational trial (ClinicalTrials.gov ID: NCT04846933) which received approval from the Ethics Committee of the Hospital District of Southwest Finland (VARHA/28314/13.02.02/2023) and is being conducted in accordance with the ethical principles outlined in the Declaration of Helsinki (WMA). Patients were recruited and treated at Turku University Hospital, Finland, with either primary debulking surgery followed by an average of six cycles of platinum–taxane chemotherapy or NACT. For NACT-treated patients, the initial step was a diagnostic laparoscopic procedure for tumor sampling, followed by an average of three cycles of NACT with carboplatin and paclitaxel. Subsequently, interval debulking surgery was performed for NACT-treated patients, aiming for complete cytoreduction followed by adjuvant chemotherapy. All patients participated in the study voluntarily and provided written informed consent.

### DNA Processing and Mutation Calling

Fresh tumor samples were collected from laparoscopy, debulking surgery, as well as from drained ascites and pleural fluids. Blood samples were also obtained as matching normal controls from each patient. Samples that met the necessary DNA content and quality criteria were forwarded to BGI or Novogene for library preparation and sequencing. WGS was carried out using either DNBSEQ (BGISEQ-500 RRID: SCR_017979 or MGISEQ-2000 RRID: SCR_017980), HiSeq X Ten (RRID: SCR_016385), or NovaSeq 6000 (RRID: SCR_016387) platforms, producing 100 bp or 150 bp paired-end reads. All samples in the validation cohort were sequenced with NovaSeq 6000 in Novogene.

We processed data in the Anduril2 workflow platform (44). DNA sample analysis included quality control, alignment to the reference human genome (GRCh38), deduplication, and estimation of cross-sample contamination, as in our previous study (3). We called somatic and germline short variants according to the validation set from the same study, whereas germline allele frequencies in tumor samples were quantified using GATK (v4.1.9.0, RRID: SCR_001876) forced in joint calling mode.

### Copy Number Variation Calling

We derived copy number segmentation using GRIDSS and the Hartwig Medical Foundation toolkit. The pipeline was constructed on the Nextflow platform (45). We called breakpoints using GRIDSS (v2.13.2, RRID: SCR_027130; ref. 46), excluding regions found in both the ENCODE (RRID: SCR_006793; ref. 47) and in-house built DECIDER blacklists (both visualized in GenomeSpy). Subsequently, GRIPSS (v2.0; ref. 48) filtered the breakpoints leveraging a panel of normals made of blood samples collected from DECIDER patients and a Dutch population. We used AMBER (v3.8, RRID: SCR_027131) to calculate B-allele frequencies—using heterozygous biallelic loci from GATK—and COBALT (v1.12, SCR_027132) to assess read depth and normalize for GC content. For all the tools we used, we used the default settings.

We then used the filtered breakpoint calls, B-allele frequencies, read depth, and somatic single-nucleotide variants as input for PURPLE (v3.7.2, RRID: SCR_022999; ref. 49). PURPLE estimated copy number segmentation profiles as well as tumor purity and ploidy of the samples. Purity estimates were corrected to zero when the difference between the maximum and minimum purity values exceeded 0.5.

Finally, we used Linx (v1.22, RRID: SCR_027133; ref. 48) with default settings to annotate and classify structural variants, using PURPLE results as input. Additional parameters included ENSEMBL gene data (RRID: SCR_002344), driver gene panel, known gene fusions, long interspersed nuclear elements, and fragile sites from Hartwig Medical Foundation resources. A schematic presentation of the pipeline is shown in the Supplementary Notes.

### Signature Extraction Pipeline

Signatures were extracted using SigProfilerExtractor (v1.1.21, RRID: SCR_023121; ref. 19) with suggested parameter settings. We selected samples for extraction based on their purity and tissue origin. The purity threshold was set to 0.20 because samples with purity below this value had fewer assigned signatures than those with higher purity (Supplementary Notes). Moreover, to prevent patients with multiple samples from having undue influence while preserving tissue variability, we retained only the highest-purity sample per tissue area for each patient when available. Tissue areas were defined as follows: primary area (fallopian tube and ovary), proximal metastases (omentum, mesentery, and peritoneum), ascites, and other (lymph nodes, bowel, vagina, uterus, and pleural fluid). Using the extracted signatures, we assigned signature activities to all remaining cohort samples that met the purity threshold using sigProfilerAssignment (v0.0.30, RRID: SCR_026899; ref. 50) with default settings.

### Complex Structural Variants

We used JaBbA (v1.1, RRID: SCR_027134) and Gurobi (v11.0; ref. 51) to identify complex structural variants, using breakpoints, read depth profiles, and the merged blacklist as input. The resulting junctions were analyzed using the gGnome R package (v1.0, RRID: SCR_027150; ref. 24), which builds a genome graph for each sample (nodes represent DNA sequences and edges represent adjacencies) and extracts structural variant classes. We annotated the number of events for each structural variant class and used Spearman correlation (with FDR correction) to check its relationship with signature activities. Because of the low maximum number of events per sample, pyrgos, tyfonas, and BFB cycle classes were converted into discrete variables (presence or absence in the sample). We then tested their enrichment in the SCN signatures using the Mann–Whitney U-test with FDR correction.

### RNA Processing

RNA-seq was performed using BGISEQ-500 (RRID: SCR_017979), MGISEQ-2000 (RRID: SCR_017980), HiSeq X Ten (RRID: SCR_016385), HiSeq 2000 (RRID: SCR_020130), HiSeq4000 (RRID: SCR_016386), or NovaSeq 6000 (RRID: SCR_016387) as 100 bp or 150bp paired-end sequencing. We then processed bulk RNA-seq reads using the SePIA pipeline (52), which consists of base trimming, alignment, gene-level count quantification, and batch-effect correction, as described in detail in our previous work (bioRxiv 2024.03.28.587131; ref. 53). Shortly, low-quality bases were trimmed using Trimmomatic (v0.33, RRID: SCR_011848; ref. 54). The trimmed reads were aligned to the GRCh38.d1.vd1 genome with GENCODE (v25, RRID: SCR_014966; ref. 55) annotations using STAR (v2.5.2b, RRID: SCR_004463; ref. 56), allowing up to 10 mismatches. Gene-level counts were quantified with eXpress (v1.5.11, RRID: SCR_006873; ref. 57), and batch effects were corrected using the POIBM method (RRID: SCR_027151; ref. 58). Subsequently, we decomposed the bulk RNA-seq data using the PRISM framework (RRID: SCR_027136; refs. 53, 58), which enabled the extraction of sample composition, scale factors, and cell type–specific whole-transcriptome profiles.

### Single-cell RNA Sample Preparation and Processing

Tumor specimens were collected from patients undergoing laparoscopy and interval debulking surgery and were then incubated overnight to generate single-cell suspensions. The scRNA-seq libraries were subsequently constructed using Chromium Single-Cell 3′ Reagent Kit v2.0 (RRID: SCR_024537, 10× Genomics) and sequenced on Illumina HiSeq 4000 (RRID: SCR_016386), HiSeq 2500 (RRID: SCR_016383), and NovaSeq 6000 (RRID: SCR_016387) sequencing instruments. We processed the fastq files using the Cell Ranger software (v6.0.1, RRID: SRC_017344), which includes steps for demultiplexing, alignment, barcode filtering, and quantification of unique molecular identifiers. The reference index was created using the GRCh38.d1.vd1 genome with GENCODE v25 annotation, and the filtered feature-barcode matrices were preprocessed using Seurat (v4.0.1, SCR_016341) toolkit (59). Cells having more than 20% of unique molecular identifier counts originating from mitochondrial genes were filtered out. scRNA-seq analyses (Supplementary Notes) were performed on a cohort of 33 samples from 25 patients; however, scaling and normalization were applied to the full dataset of 95 samples from 57 patients (bioRxiv 2024.03.28.587131, bioRxiv 2025.06.13.659489; refs. 53, 60–62).

## Supplementary Material

Supplementary Notes 1 Additional detail description of the methods used in the main text

Supplementary Table S1 Associated publication for each single-cell RNA sample.

Supplementary Figures S1-S9 Supp. Fig. S1 shows signature robustness. Supp. Fig. S2 displays the association of all signatures with homologous recombination and BRCA1/2 mutations. Supp. Fig. S3 depicts the association of signatures with whole genome duplication, amplification of CCNE1, KRAS, MYC, and MECOM and the relationship with survival. Supp. Fig. S4 shows how complex structural variants manifest and in which signatures they are prevalent. Supp. Fig. S5 explains the SCN-G segmentation aberration. Supp. Fig. S6 presents the validation dataset results in terms of survival and genomic characteristics. Supp. Fig. S7 displays transcriptomics analysis results for the proliferative HRP subtype. Supp. Fig. S8 depicts the transcriptomic results for the EMTHRP subtype. Supp. Fig. S9 contains the results from the transcriptomic analysis for the core HRP subtype.

## Data Availability

All raw DNA sequencing data are submitted to the European Genome-phenome Archive (EGA) and will be publicly available under study accession number EGAS00001006775. Raw bulk RNA-seq data are deposited in the EGA and are publicly available (EGAS00001004714). WGS data from cell lines were gathered from Sequence Read Archive (accession number PRJNA523380). Published raw scRNA-seq data are indicated in Supplementary Table S1; unpublished samples have been deposited in the EGA under the study accession number EGAS00001005010. Two of eight organoids are deposited in Auria Biobank (https://www.auria.fi/biopankki/en/). Panel-of-normal data for mutation calling included The Cancer Genome Atlas normal samples, which were available through the Database of Genotypes and Phenotypes. The main steps of the analysis are available on GitHub (github.com/HautaniemiLab/CIN-subtypes) and CodeOcean (codeocean.com/capsule/8597092/tree/v1).
