# Supplementary material for: Decoding the Genomic and Functional Landscape of Emerging Subtypes in Ovarian Cancer
Source: Cancer Discov. 2025 Jul 31;15(11):2262–77. doi: 10.1158/2159-8290.CD-25-0652 (PMC12580789; doi:10.1158/2159-8290.CD-25-0652)
Supplement: Supplementary Notes 1 — Additional detail description of the methods used in the main text [file cd-25-0652_supplementary_notes_1_suppsd1.pdf]

Supplementary Notes for:

Decoding the Genomic and Functional Landscape of Emerging Subtypes in Ovarian Cancer

Giulia Micoli[1], Kari Lavikka[1], Yilin Li[1], Anna Pirttikoski[1], Daria Afenteva[1], Wojciech Senkowski[2], Giovanni Marchi[1], Anna Vähärautio[1], Taru A. Muranen[1], Titta Joutsiniemi[4], Sakari Hietanen[4], Anni Virtanen[3], Krister Wennerberg[2], Johanna Hynninen[4], Jaana Oikkonen[1], Sampsa Hautaniemi[1*]

[1]Research Program in Systems Oncology, Research Programs Unit, Faculty of Medicine, University of Helsinki, Helsinki, Finland,

[2]Biotech Research & Innovation Centre (BRIC), University of Copenhagen, Copenhagen, Denmark;

[3]Department of Pathology, University of Helsinki and HUS Diagnostic Center, Helsinki University Hospital, Helsinki, Finland;

[4]Department of Obstetrics and Gynecology, University of Turku, and Turku University Hospital, Turku, Finland.

Corresponding author:

Sampsa Hautaniemi (sampsa.hautaniemi@helsinki.fi)

Contents

1 Feature selection ... **3**
1.1 Copy number features ... 4
1.2 Structural variation features ... 6

2 Signature validation ... **8**
2.1 Sequencing platforms ... 8
2.2 Parameter-variation test ... 9
2.3 Extension to GATK-ASCAT segmentation ... 10

3 Genomic statistical analyses ... **11**
3.1 Statistical tests ... 11
3.2 Mutational signatures ... 11
3.3 Homologous Recombination Deficiency (HRD) analysis ... 12
3.4 GISTIC analysis and region selection ... 13
3.5 Driver gene amplifications and mutations ... 14
3.6 Ploidy, Whole Genome Duplication, and LOH ... 14
3.7 Complex structural variants analysis ... 15

4 Signature activities clustering and analysis ... **16**
4.1 Clustering ... 16
4.2 Patients' stability ... 17
4.3 Genomic characterization ... 18
4.4 Clinical analysis ... 19
4.5 Transcriptomic analyses ... 20
4.6 Cell lines characterization for drug sensitivities ... 24
4.7 Organoids ... 25

5 Validation ... **26**
5.1 Genomic comparison ... 26
5.2 Survival analysis ... 27

6 References ... **28**

1 Feature selection

The data processing workflow was carried out as outlined in Note Figure 1, ensuring a systematic and reproducible approach to data analysis.

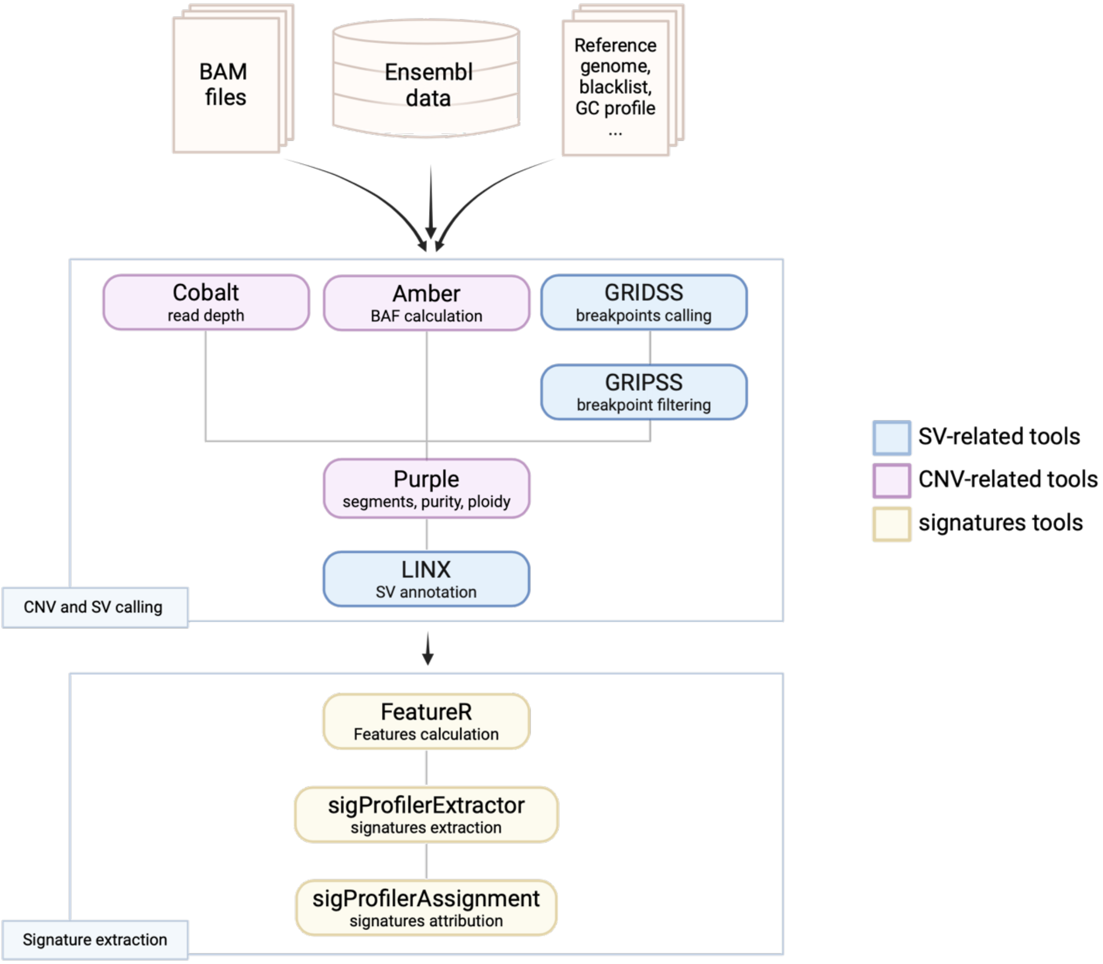

Note Figure 1: Schematic representation of the pipeline. Inputs for the pipeline, including BAM files for normal samples and tumors, ENSEMBL data and additional material feed the first part of the pipeline for SV and CNV calling. The results from the first part are then used to quantify features and extract signatures.

After structural variation (SV) and copy-number (CN) calling, we performed feature selection to identify the most relevant genomic alterations for downstream analyses.

To fully capture chromosomal instability, we reviewed various CN and SV signature approaches from the literature. Indeed, CN signature recognition has advanced, but consensus on methods and interpretation remains unclear. The scientific community has developed various methods for identifying CN and SV signatures, but no definitive standard has emerged. Early CN signature methods were constrained by low-resolution data [1–3], while existing SV or rearrangements signature approaches [4,5] often use a limited set of features or apply broadly across cancer types, grouping ovarian cancer patients into uninformative categories. To

overcome these limitations, we combined existing CN with new SV ones for a more comprehensive analysis.

To ensure robust signature attribution, we applied a 20% purity threshold, since we observed a significant drop in the number of attributed signatures when sample purity fell below this level (Note Figure 2).

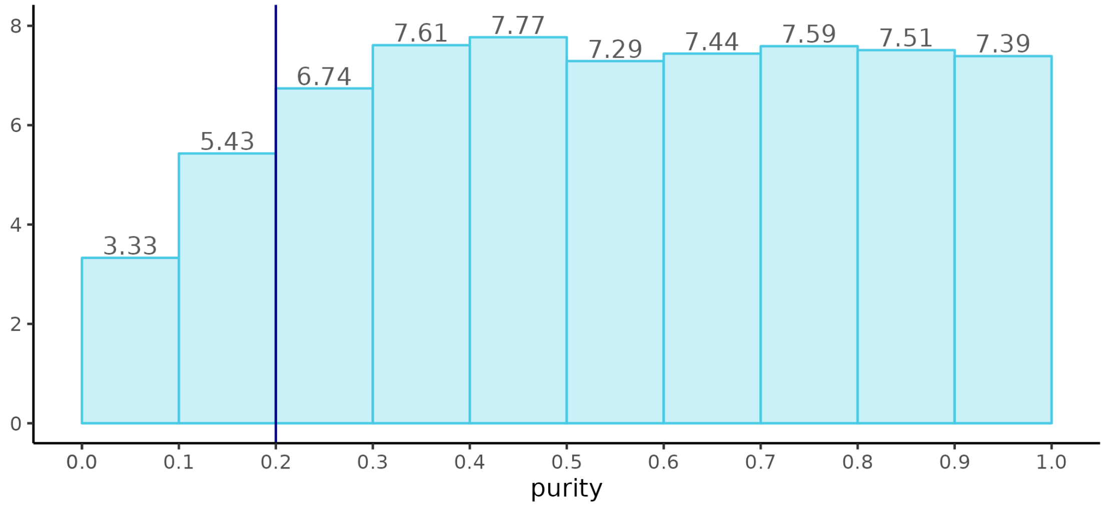

Note Figure 2: Histogram of the purity values of the entire cohort. In each bin we show the average number of signatures attributed to corresponding samples. The threshold has been set to 0.2, where there is a clear drop in the number of attributed signatures.

1.1 Copy number features

We retained the features from Drews *et. al* 2020 [2] because they effectively capture multiple events important for identifying biological processes. While we followed the original calculation methods, we adapted the discretization approach to accommodate our different data type (WGS instead of shallow WGS). Specifically, we experimented with various discretization techniques [2,3] and ultimately selected Jenks natural breaks (BAMMtools, v2.1.12, RRID: SCR_027137) for all features, except for 5Mb breakpoints, where we opted for Poisson mixture models (flexmix R package, v2.3-20, RRID: SCR_027152). This approach allowed us to meaningfully partition the data (Table1), as demonstrated in Note Figure 3.

Table1: copy number features discretization breaks.

Segment size		Breakpoints per chromosomal arm		Changepoint		Oscillation length		Breakpoints per 5MB	
**Group**	**Break**	**Group**	**Break**	**Group**	**Break**	**Group**	**Break**	**Group**	**Mean**
Segsize1	1.97 kb	bpArm1	4	Chg1	0.57	Osc1	1	fiveMB1	0
Segsize2	375 kb	bpArm2	12	Chg2	1.32	Osc2	3	fiveMB2	3.04
Segsize3	149 Mb	bpArm3	31	Chg3	2.58	Osc3	6	fiveMB3	11.05
		bpArm4	587	Chg4	6.62	Osc4	75	fiveMB4	27.66
				Chg5	138.62			fiveMB5	94.46

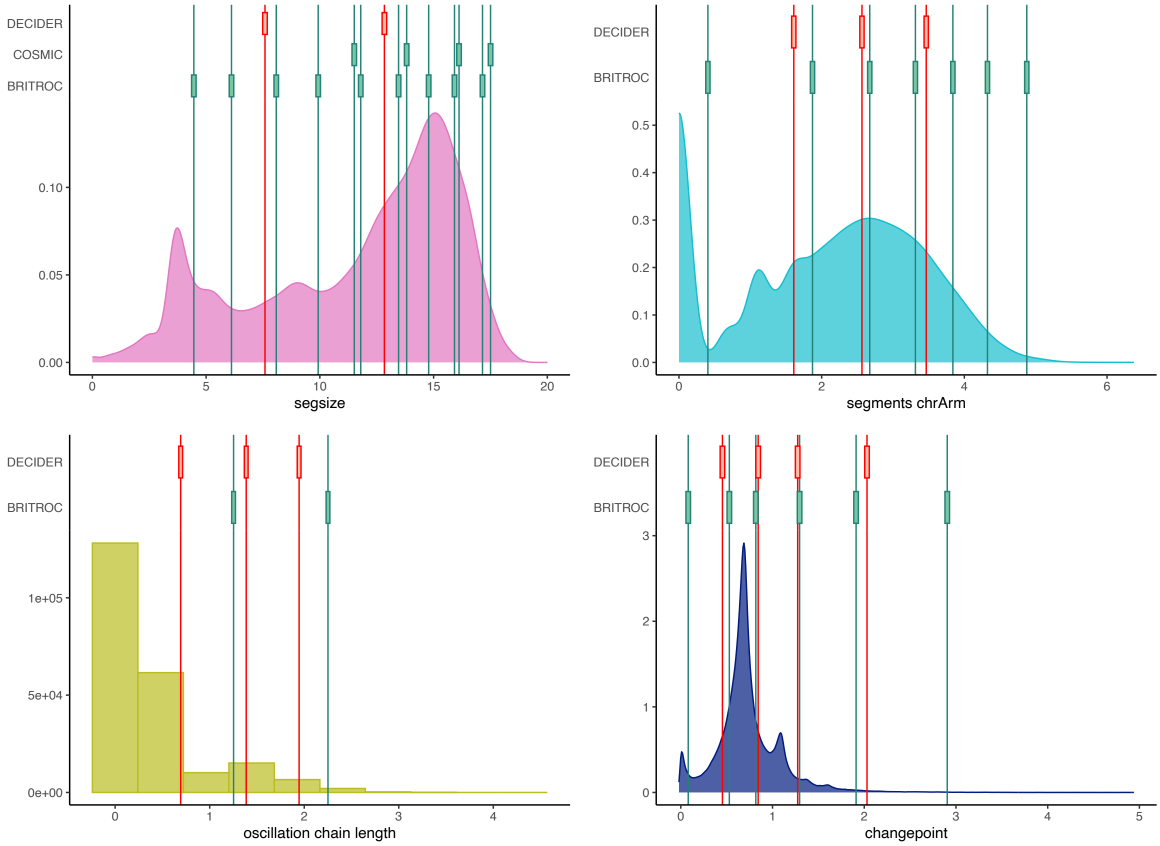

Note Figure 3: Distribution in a logarithm scale of CN-related features for which the discretization method differs from [12]. The distribution color matches the classes displayed in main Fig. 1. Red lines represent the division adopted in our method and are produced with Jenks natural breaks algorithm. The dark green lines represent the previously adopted discretization thresholds used in literature [2], [3].

1.2 Structural variation features

Features describing SVs in literature do not comprehensively describe the category. Thus, to calculate meaningful features, we used the annotations from the tool LINX (v1.22, RRID: SCR_027133). LINX resolves variants into clusters, that are groups of variants forming an event that transforms the genome from a stable state to another stable state [6].

1.2.1 Simple and complex events

Simple events are clusters constituted by one or two junctions that form a local deletion, tandem duplication or unbalanced translocation. Complex events comprise events involving more junctions and include reciprocal inversions or translocations, templated insertions, insertions, double minutes, and other complex clusters. The number of these events is extracted and used as two distinct features.

These categories are important since the prevalence of one of the two groups can identify biological processes. For example, the prevalence of simple events can indicate homologous recombination deficiency, and a prevalence of more complex events can indicate a more sophisticated process.

1.2.2 Inter-chromosomal and intra-chromosomal events

For each variant, the two breakends are extracted and the pairs lying on the same chromosome are classified as intrachromosomal while the ones in different chromosomes as intrachromosomal. Their counts in each sample constitute the corresponding features.

These features help in the identification of distinct mechanisms since intrachromosomal SVs arise mainly from replication errors, non-allelic homologous recombination or break-induced replication, while inter-chromosomal SVs often result from chromosomal breakage and repair.

1.2.3 Compact and sparse events

Events with more than three junctions on the same chromosome are selected and analyzed for genomic location. First, we calculated the distance between each pair of breakpoints and then we classified them in sparse and compact by applying two gaussian mixture models (R package Mclust v6.1.1 [7], RRID: SCR_027153 , Table2). The count of compact and sparse events is then reported.

This is important since clusters of SVs within specific regions of the chromosome can indicate areas of genomic instability, often associated with specific mechanisms of DNA damage and

repair. For example, regions with high densities of SVs might be hotspots for replication errors or areas prone to chromothripsis.

Table2: compact-sparse feature breaks.

Class	Mean	Standard deviation
compact	1.49 kb	2.53 kb
sparse	5.27 Mb	14.69 Mb

1.2.4 Deletion and duplication lengths

Simple deletions and duplications from LINX results are extracted, their lengths converted to logarithmic scale and categorized using Jenks natural breaks (getJenksBreaks function from BAMMtools, Table3). The count of each category is then recorded as a feature. The number of classes was chosen after testing multiple values and visual inspection of the distribution. Considering the lengths of these SVs is fundamental for the identification of several biological processes such as *CDK12*mut, *BRCA1*mut and *BRCA2*mut patterns.

Table3: deletion and duplication length feature breaks.

Deletion length		Duplication length	
Group	Range	Group	Break
Del1	0-201b	Dup1	0-1.01kb
Del2	202b-2.53kb	Dup2	1.01kb-45.2kb
Del3	2.53kb -36.06kb	Dup3	45.2kb-1.52Mb
Del4	36.06kb-1.13Mb	Dup4	> 1.52Mb
Del5	> 1.13Mb		

1.2.5 SV categories

Resolved SV events are retrieved from LINX results and summarized in the following classes:

- Insertions: category = SIMPLE, resolvedType = INS
- Reciprocal inversions: category = RECIPROCAL, resolvedType = RECIP_INV_DEL_DUP, RECIP_INV_DUPS, RECIP_INV_DELS, RECIP_INV
- Reciprocal translocations: category = RECIPROCAL, resolvedType = RECIP_TRANS_DEL_DUP, RECIP_TRANS_DUPS, RECIP_TRANS_DELS, RECIP_TRANS
- Unbalanced translocations: category = SIMPLE, resolvedType = UNBAL_TRANS
- LINE transposons: category = INVERSIONS, resolvedType = LINE

The number of chromothripsis events is derived instead from the R package ShatterSeek (v1.1, RRID: SCR_026463) using the segmentation from PURPLE (v3.7.2, RRID: SCR_022999) and the SV calls from GRIDSS (v2.13.2, RRID: SCR_027130) as input. The extracted chromothripsis clusters are then classified as “high-confidence” and “low-confidence” according to the recommendations [8]. The number of high-confidence events constitutes the final feature.

1.2.6 Quantiles

The variable logR derived from PURPLE segmentation and is calculated as the logarithm of the segment copy number over the sample ploidy:

$$\log(R) = \log\left(\frac{copyNumber}{ploidy}\right)$$

This value represents the difference in copy number of each segment with respect to the ploidy of the sample, allowing to account for conditions that differ from the normal diploid state. For each sample, the 80th and 20th quantiles of log(R) are calculated. To ensure these values are positive, 1 unit is added to both, and the 20th quantile's sign is reversed. These adjusted values serve as the features *dup_magnitude* and *del_magnitude*, respectively. Duplication and deletion magnitudes allow to determine if the genome presents a high number of alterations (duplications or deletions), hence regions with an increased amount of genomic instability.

2 Signature validation

Extracted signatures were tested for their reliability with three experiments:

- Concordance with different sequencing platforms
- Multiple extraction after parameter variation
- Application to another CN segmentation pipeline

2.1 Sequencing platforms

After signature extraction and attribution to the entire cohort, we evaluated the consistency of signature activities across 13 duplicated samples sequenced on different platforms. The platforms used are Novaseq (RRID: SCR_016387) and HiSeq from Illumina (RRID: SCR_016385), and DNBSEQ from BGI (BGISEQ-500 RRID: SCR_017979, MGISEQ-2000 RRID: SCR_017980).

Signature activity values and cosine similarity between the duplicated samples are displayed in Extended Data Fig.1a. We observed strong coherence in the analysis, demonstrating that the

process is neither biased nor sensitive to differences in sequencing platforms, thereby affirming its robustness.

2.2 Parameter-variation test

To further test the stability of the procedure, we used a set of 50 random samples from the entire cohort, and we performed rounds of signature extraction with sigProfilerExtractor [9](v1.1.21, RRID: SCR_023121), varying the parameters setting. In each of the 32 rounds, only one parameter deviated from the default settings, ensuring that each parameter was both minimized and maximized across different rounds. In each extraction, we used the seed of the run with default settings. This approach allowed for a comprehensive assessment of the impact of individual parameters on the signature extraction process.

After that, all extracted signatures have subsequently been analyzed for their similarity. First, we compared the number of extracted signatures per each round, which was stable at 7 with some variation in a few cases (Note Figure 4. Subsequently, groups of matching signatures were identified by finding the highest cosine similarity to the reference run (conducted with default settings), which defined the reference group. Signatures within the same matching group were then compared by calculating the variance for each feature and evaluating the cosine similarity across all runs. The results from the comparison are shown in Extended Data Fig.1b.

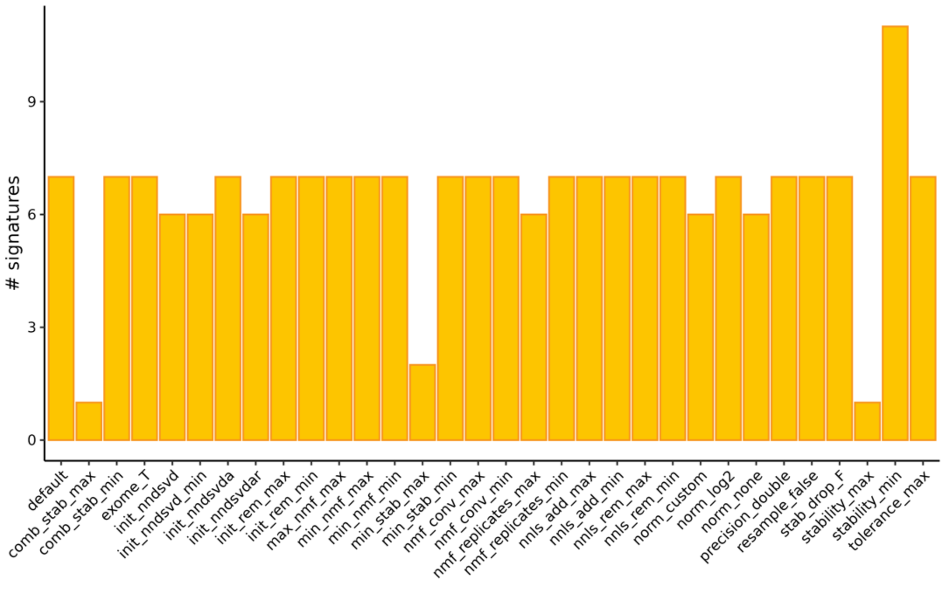

Note Figure 4: number of signatures per each extraction round.

2.3 Extension to GATK-ASCAT segmentation

To prove the importance of SVs in the extraction of signatures and to verify the expandability of the method to other tools that do not provide SV calling, we adapted and applied the extraction to another CN segmentation. The pipeline used for segmentation and purity-ploidy estimation is built upon Anduril2 platform [10] and employs GATK (established best practices, v4.1.9.0, RRID: SCR_001876), which utilizes only read-depth and BAF, and a reimplemented form of ASCAT algorithm [11] (RRID: SCR_016868) as described in [12].

The extraction cohort comprises the same samples of the original work, but it used only the features computable from this segmentation (CN features, duplication and deletion magnitudes and duplication lengths), with a total of 24 features. Duplications were estimated from segments CN, particularly a segment is considered a duplication if it matches all the following conditions:

- its total copy number is higher than the copy number of the flanking segments
- previous and next segment copy numbers are equal
- its loss of heterozygosity (LOH) is higher than LOH of flanking segments

The LOH is calculated as $LOH = 2 \cdot |BAF - 0.5|$ where B allele frequency is already corrected for purity and ploidy.

The 11 obtained GATK-ASCAT (GA) signatures were first matched with the original SCN signatures. From the original SCN signatures definition weights, unused features were removed and then cosine similarity was calculated between each pair of GA and SCN signature definitions revealing that the matching pairs were almost identical (average cosine similarity of matching pairs equal to 1). Subsequently, GA signatures were estimated in the entire cohort and the resulting activities correlated to the activities of SCN signatures. For each matching pair, the distribution of activities has been compared in Extended Data Fig. 1c and in Note Figure5, showing that they are very different in most matching pairs with very low Spearman correlations. This analysis highlights the critical role of SV features in the efficacy of our signature extraction methodology. Exclusion of these features compromises the practical application of the signatures.

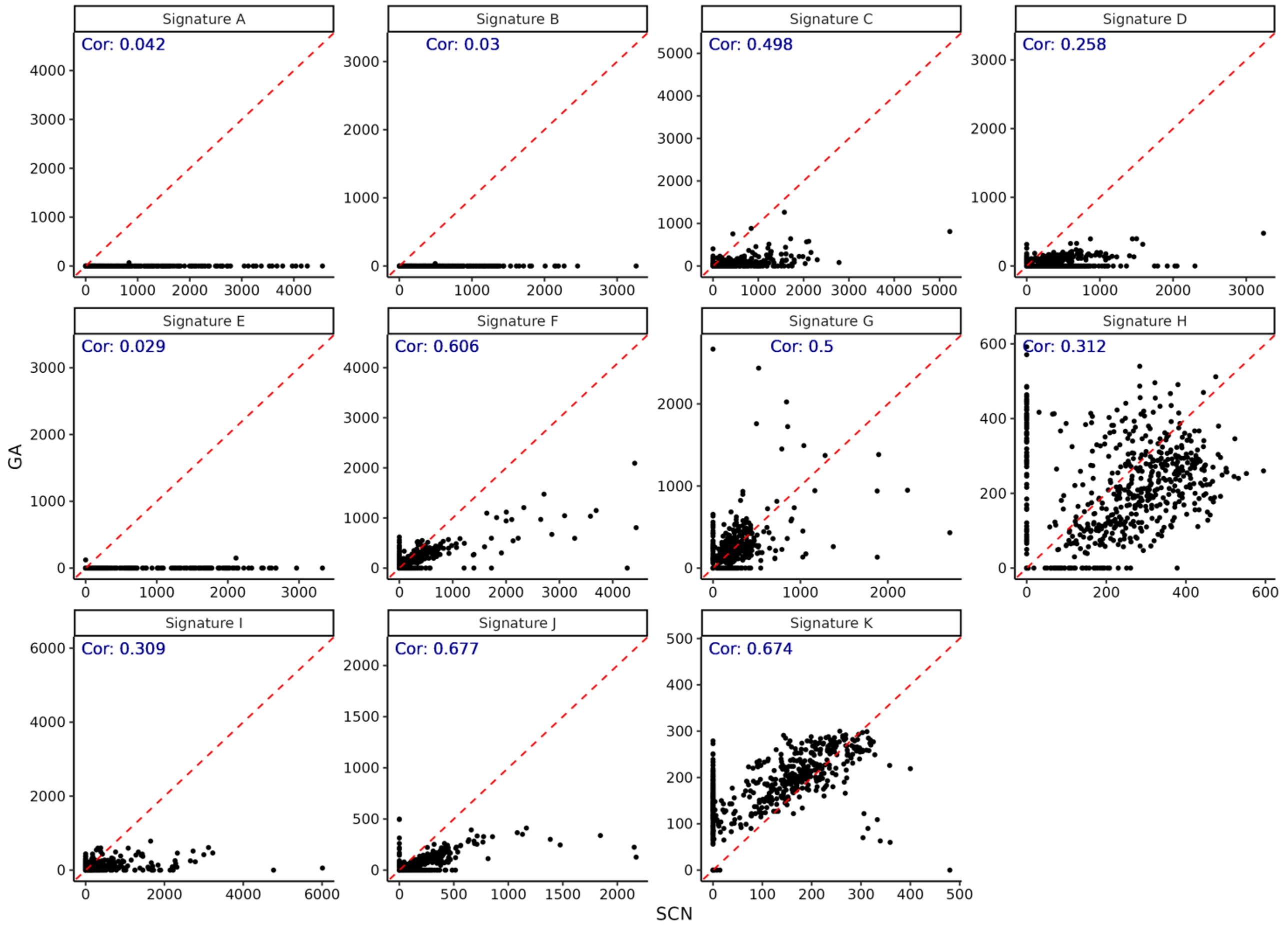

Note Figure 5: comparison of signature activities between GATK-ASCAT (GA) and SCN signatures. In each scatterplot, the bisector (red dashed line) and the Spearman correlation are reported.

3 Genomic statistical analyses

3.1 Statistical tests

All statistical tests conducted aimed to compare the signatures to a categorical variable were done by applying the Mann-Whitney U-test and then correcting for multiple hypothesis testing using FDR. Significance threshold was set to 0.05.

3.2 Mutational signatures

Short-mutation signatures were downloaded from the COSMIC database [13]. COSMIC (v3.3.1, RRID: SCR_002260) single-base substitution (SBS) and double-base substitution (DBS) were adjusted for GRCh38 nucleotide frequencies excluding chromosomes Y and M. These and indel (ID) signatures were fitted with a multi-step approach as described for variant clusters in Lahtinen et al [14]. Variant filters were applied accordingly. The final sample attribution step was

restricted to backward selection with a threshold of 0.01. Signatures reported in > 20% of ovarian cancers in COSMIC were selected as starting signatures.

Mutational signature and SCN signature activities were compared using Spearman correlation with significance corrected for multiple testing using FDR.

3.3 Homologous Recombination Deficiency (HRD) analysis

Mutational homologous recombination (HR) status was determined based on samples SBS3 signature activity [15,16]. Patients were classified as mutational HRD if all their samples showed SBS3 activity >0; otherwise, they were considered HR-proficient (HRP). We compared signatures activities between HRD and HRP samples to study the association of signatures with the mutational HR status (Extended Data Fig. 2a).

To further differentiate types of HRD, we investigated the associations with *BRCA1* and *BRCA2* mutations. Germline and somatic mutations in *BRCA* genes were identified through the manual curation of mutation-calling pipeline results. The prevalence of signature activities in *BRCA1* and *BRCA2* mutated samples was analyzed both across the entire cohort (Extended Data Fig. 2b-c) and specifically within HRD patients. Table4 provides a summary of the patients carrying somatic and germline *BRCA1*, *BRCA2* mutations and mutations in *RAD51c* and *RAD51d*.

Table4: homogeneous somatic and germline mutations in HR-related genes used for the association analysis.

GENE	GERMLINE	SOMATIC
BRCA1	6	17
BRCA2	5	5
RAD51C	9	1
RAD51D	3	0

Patient-level summarization involved filtering of samples to include only primary-phase samples with an SCN-G signature fraction below 0.4. The threshold was determined using the Jenks natural breaks method, applied to the entire cohort's SCN-G values with three breaks. Signature activities were averaged per patient using the median across samples. Each signature was then binarized in high and low activity using the overall patient median as a threshold.

To determine whether signatures associated with HRD could serve as predictors of HR status, various predictive models were constructed in the R environment (R packages caret v7.0.1 RRID: SCR_022524, car v3.1.3 RRID: SCR_022137). The mutational HR status derived from

COSMIC SBS3 signature was used as reference. We divided the dataset into a training set (80% of patients) and a test set (20%) and then trained multiple logistic regression models with 10-fold cross-validation incorporating different sets of signatures. Predictive capacity and ROC curves were then evaluated on the test dataset (Extended Data Fig. 3a). The model with the best predictive power was the one utilizing only the HRD-related signatures SCN-A, SCN-B and SCN-E.

The link between HRD signatures and clinical response was analyzed using Kaplan-Meier curves, based on platinum-free interval (PFI) and cancer relapse (R packages survminer v0.5.0 RRID: SCR_021094, survival v3.8.3 RRID: SCR_021137). In addition to the HRD signatures, we found that the SCN-I, SCN-J, and SCN-D signatures are associated with significantly poorer survival outcomes, as they predominantly identify HRP patients in contrast to HRD (Extended Data Fig. 3b).

3.4 GISTIC analysis and region selection

Driver CN events in the cohort were identified by retrieving regions with significant deviations from reference copy numbers using GISTIC (v2.0.23, RRID: SCR_000151) with default parameters [17]. The input data included PURPLE segmentation of each patient's highest-purity sample. We then selected the highest amplification and deletion peaks from each chromosome arm among the detected regions. Next, we extracted for each sample the segments falling in the peak regions: if multiple segments fell within the amplified peaks, we took the segment with the highest copy number for amplified peaks, and the one with the lowest copy number for deletion peaks. These selected segments were then classified based on CN/ploidy ratios as amplification (CN/ploidy ≥ 2.30), gain (CN/ploidy ≥ 1.39), or normal. Similarly, for deleted regions, segments were classified as deleted if their CN/ploidy ratio was ≤ 0.78. Classification thresholds were established by applying the Jenks natural breaks optimization method with four centers to samples without whole genome duplication (WGD, see chapter 3.6, Note Figure 6).

We then investigated signature prevalence in samples with amplified or gained regions: for each amplification peak, samples were divided into amplified and not amplified (gained and not gained), and the difference of signature activities was then compared. The same analysis was conducted for deletion peaks.

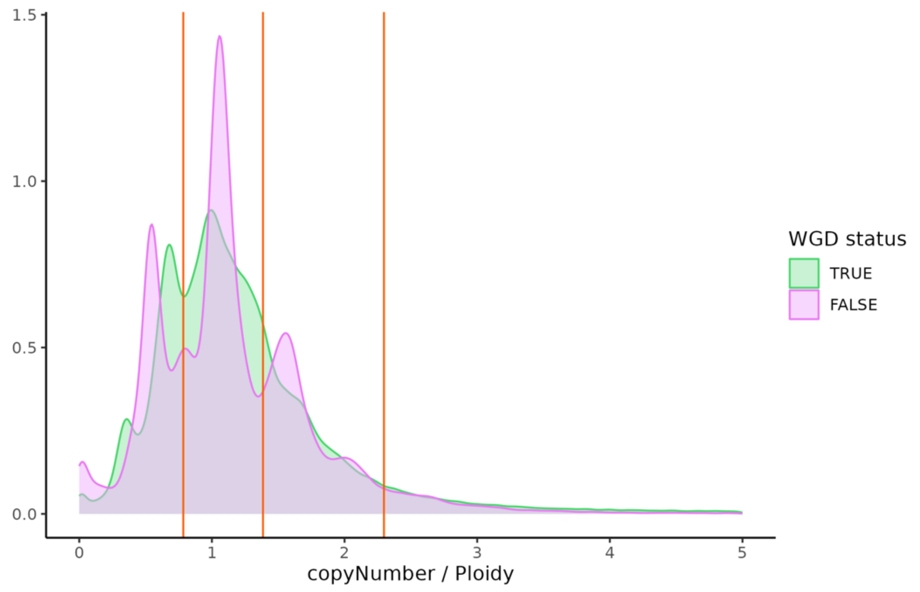

Note Figure 6: density plot of the copyNumber/ploidy value distinguished between WGD samples (green) and normal samples (pink). Orange lines represent the threshold calculated using Jenks natural breaks algorithm, defining deletion, gain and amplification of a segment.

3.5 Driver gene amplifications and mutations

We explored the correlation and association of signatures with commonly mutated or amplified ovarian cancer genes, focusing particularly on the loss-of-function mutations in *CDK12*, and *NF1*, as well as the amplification of *CCNE1*, *KRAS*, *MYC*, and *MECOM*. Copy number values for driver genes were defined using the minCopyNumber field from the PURPLE output (gene copy-number file). First, we estimated the relationship between driver genes CN and signature activities using Spearman correlation with significance corrected for multiple testing using FDR. Next, in each sample, driver genes were classified as amplified if the CN/ploidy ratio was ≥ 2.30 and present with more than 8 copies, as described in the previous chapter. Somatic mutation calls for *CDK12* and *NF1* were manually curated by geneticists. We then statistically compared signature activities in samples with amplified or mutated drivers.

3.6 Ploidy, Whole Genome Duplication, and LOH

To test any association of signatures with high values of ploidy and genome doubling we first calculated the correlation between signature activities and ploidies from PURPLE (Note Figure 7). To have a better understanding, we then split ploidy values into two groups (low-ploidy and high-ploidy) using Jenks natural breaks algorithm (threshold at 2.68). compared the activities for each signature between high and low ploidy samples, revealing SCN-C (p = 1.04e-33), SCN-I (p = 1.59e-39) and SCN-J (p = 1.38e-10) significantly linked to high ploidy.

WGD status was calculated as in [18], first computing the mean LOH and mean ploidy per sample using integer segment CN weighted by segment length and purity, and then estimating the presence of WGD if

$$WGD = 2.9 - 2 \cdot meanLOH \ \leq meanPloidy$$

Association between signatures and WGD was then tested as in 3.1.

We also wanted to test any possible link to loss of heterozygosity (LOH). The LOH level for the entire genome was estimated as:

$$LOH = \frac{\sum(2 \cdot |segmentBaf - 0.5|segmentLength)}{\sum segmentLength}$$

and the threshold for defining high/low level of LOH was defined using k-means. The difference in signature activities between LOH high and low samples was tested as above.

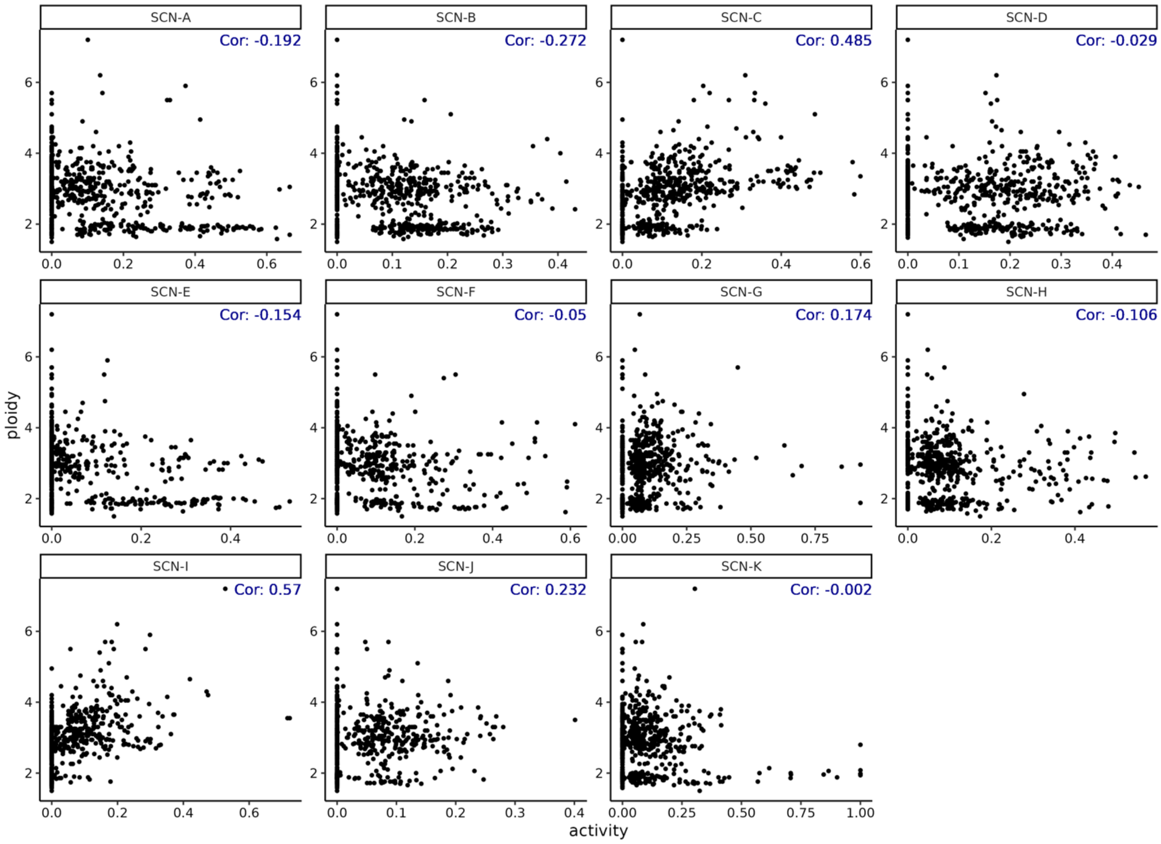

Note Figure 7: Spearman correlation between signature activities and ploidies.

3.7 Complex structural variants analysis

The extraction of complex SVs was performed by using JaBbA (v1.1, RRID: SCR_027134) [19] and the optimization software Gurobi (v11.0) [20] which used as input the SVs derived from PURPLE, the read depth profile from COBALT and the merged blacklist. The resulting junctions were analyzed with gGnome R package (v1.0, RRID: SCR_027150) [19], which for each sample first

builds a genome graph (nodes represent DNA sequences and edges adjacencies), then extracts SV classes.

We annotated the number of events for each SV class and calculated the Spearman correlation with signature activities (FDR correction). Given the low maximum number of events per sample, the interesting classes (pyrgo, tyfona and BFB cycles) were transformed into discrete variables (presence or absence in the sample) and tested for the enrichment in the different SCN signatures. After the quantification analysis, we examined the genomic distribution of each class of complex structural variants:

- Intra-chromosomal events (such as pyrgo): we annotated the chromosome for each event and then, after normalizing for chromosome length, compared the differences between chromosomes pairwise.
- Inter-chromosomal events: we annotated the number of breakpoints per chromosome for each breakpoint cluster. For BFBs and chromoplexy, we identified the most common links between chromosomes, while for tyfonas, we determined the center of each cluster (defined as the location with the highest number of breakpoints per chromosome) and compared the chromosomes where these centers were located.

Additionally, we also analyzed the breakpoint distribution across chromosomes by dividing the genome into 0.5Mb intervals and counting the breakpoints within each. Noticing a particularly high peak on chromosome 19, we compared *CCNE1*-amplified samples with *CCNE1*-normal ones and found a significant difference in this region (main Fig. 2F), supporting our conclusion that *CCNE1* amplification is caused by BFB cycle events.

4 Signature activities clustering and analysis

4.1 Clustering

Samples were initially filtered by excluding those with SCN-G activity ≤ 0.4, resulting in a cohort of 629 samples. This cohort was then clustered based on their signature activities using ConsensusClusterPlus (v1.68, RRID: SCR_016954), which evaluated 1000 subsamples comprising 90% of the full dataset.

Hierarchical clustering was performed using Euclidean distance and the ward.D2 linkage method to ensure cluster stability. This process was repeated 50 times, with each iteration assessing the optimal number of clusters and the consistency of sample assignments. The most stable clustering resulted in five clusters; however, based on the genomic properties

explored in the next chapter, and since their stability metrics were comparable to those of the five-cluster solution, six clusters were ultimately adopted. Across the 50 runs, only 26 samples shifted between two clusters, indicative of minimal variability. The final cluster assignments were determined by selecting the most frequent assignment for each sample across all iterations.

4.2 Patients' stability

Patient stability within clusters was evaluated using two measures: general stability, which considers all available samples, and primary stability, which includes only pre-treatment samples. Patients were classified as stable if all their samples consistently fell within the same cluster. If a patient had more than five samples with only one deviating, the outlier was reassigned to the most frequent cluster, and the patient remained classified as stable. Primary stability was assigned to patients whose pre-treatment samples all belonged to the same cluster. General stability summary for each cluster and tissue composition are shown in Note Figure 8.

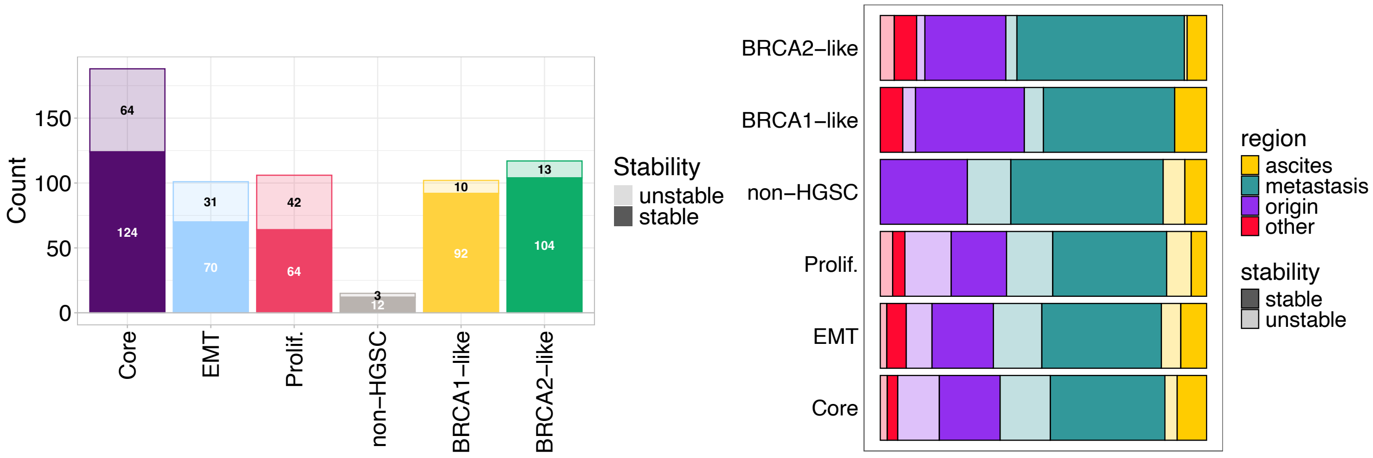

Note Figure 8: In the left panel sample composition of clusters divided in samples belonging to stable patients and unstable patients. In the right panel tissue composition of the subtypes dividing each tissue type by general stability. Origin tissues include tube and ovary, metastatic region includes peritoneum, omentum and mesentery while other region include uterus, vagina, bowel, lymph nodes, and pleural fluid.

To investigate whether tissue sampling site could influence classification stability, we analyzed the distribution of tissue types between stable and unstable patients. A chi-squared test comparing the two groups revealed no significant enrichment of any specific tissue site in either category (p = 0.25; see Note Figure9). These results suggest that the observed instability is not driven by sampling bias or tissue-specific enrichment, but rather reflects intrinsic genomic heterogeneity within certain patients.

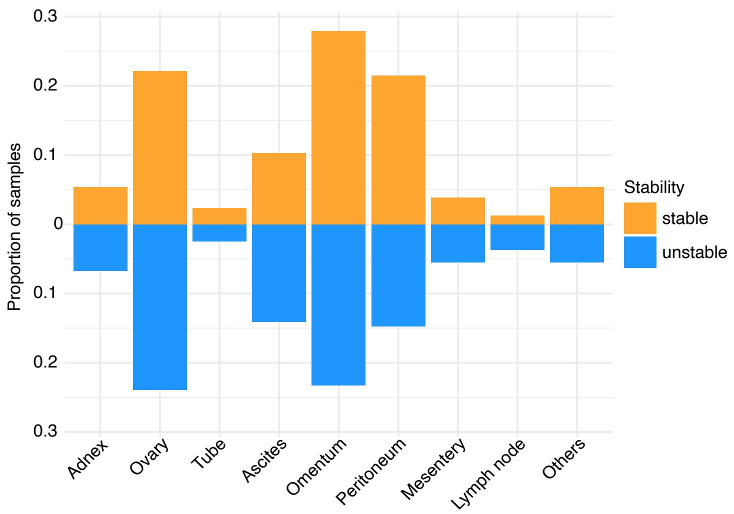

Note Figure 9: Tissue composition of stable (top bars) and unstable (bottom bars) patients. The y-axis represents the proportion of samples within each stability category. No significant enrichment is observed across tissue types. While stable patients show a slight increase in omental and peritoneal samples, and unstable patients show a slight increase in ascites and mesenteric samples, these differences are not statistically significant.

4.3 Genomic characterization

To identify mutations that characterize each subtype, we analyzed the somatic SNVs from all samples and focused on exonic and splicing mutations, excluding synonymous mutations or those with unknown function. Since our goal was to identify mutated genes rather than the total number of mutations, we filtered the mutation with the highest allele frequency for each gene. We also selected mutations that were present in all samples from the same patient and then, for each gene, we calculated the proportion of mutated patients within each subtype. The association of patients with the evolutionary state was done according to [14].

The composition of the HRD subtypes was as follows:

- BRCA2-like**:** 9 patients with *BRCA2* deficiency, 2 with *BRCA1* deficiency, 12 with *RAD51* deficiency, 2 with *BRIP1* deficiency, and 14 with no identifiable HRD-related mutation.
- BRCA1-like: 16 patients with *BRCA1* deficiency and 17 with no detectable HRD-related mutation.

Note: In main Fig.3 tumor burden and number of breaks valued for the patient EOC1106 have been set to the maximum values found in the rest of the cohort. This operation was necessary because displays a hypermutator phenotype, biasing the tumor mutational burden and number of breaks results.

4.4 Clinical analysis

Survival analysis was conducted across molecular subtypes, including only patients with primary stability and histologically confirmed HGSC, while excluding the non-HGSC cluster. Kaplan-Meier curves were generated for both overall survival (OS) and platinum-free interval (PFI) in months. Pairwise log-rank test results, adjusted using the Benjamini-Hochberg correction, are presented in Table 5 for OS and Table 6 for PFI.

Table5: log rank test with Benjamini-Hochberg p-values from the Kaplan-Meier survival analysis between clusters.

OS (months)	Core HRP	Prolif. HRP	EMT HRP	BRCA1-like
Prolif. HRP	0.78			
EMT HRP	0.78	0.72		
BRCA1-like	0.35	0.68	0.23	
BRCA2-like	7.54e-5	4.31e-4	2.12e-4	0.005

Table6: log rank test with Benjamini-Hochberg p-values from the Kaplan-Meier survival analysis between clusters.

PFI (months)	Core HRP	Prolif. HRP	EMT HRP	BRCA1-like
Prolif. HRP	0.67			
EMT HRP	0.21	0.14		
BRCA1-like	0.03	0.21	0.001	
BRCA2-like	0.001	0.01	3.74e-6	0.31

Primary chemotherapy response were assessed using RECIST 1.1 criteria [21] and categorized as bad responders (stable and progressive disease) or good responders (partial and complete response).

Comparison of HR assessment methods was conducted between ovaHRDscar [22] (v0.1.0, RRID: SCR_027138), a clinical tool for determining PARP inhibitor eligibility, and HR status assigned by molecular clusters. We assessed the concordance of the two models using the time-dependent concordance probability estimate (CPE), implemented through the phcpe function from the CPE R package (v1.6.3, RRID: SCR_027140). Cox regressions were first performed and then passed to the phcpe function. The analysis revealed slightly higher concordance values for the CIN subtypes method in both overall survival (OS: C = 0.606) and progression-free interval (PFI: C = 0.599), compared to the ovaHRDscar classification (OS and PFI: C = 0.584). These results suggest that the CIN subtypes model has a modestly better ability to predict clinical outcomes.

To better understand how the CIN subtypes model differs from ovaHRDscar, patients were classified into four categories:

- HRD by both methods
- HRD by clusters but HRP by ovaHRDscar
- HRP by clusters but HRD by ovaHRDscar
- HRP by both methods

Kaplan-Meier survival analysis was performed across these groups using PFI (main Fig.4f) and OS (Note Figure 10, left panel) as time metric.

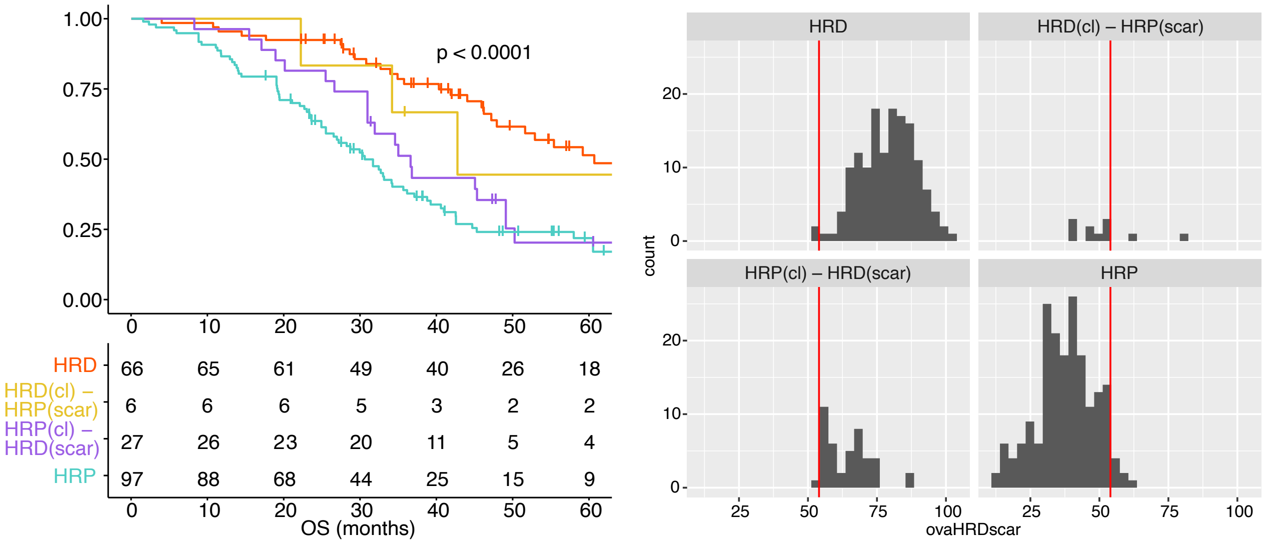

Note Figure 10: In the left panel, Kaplan-Meier survival analysis of 5 years OS comparing homologous recombination (HR) status determined by ovaHRDscar and molecular clustering (p = <0.0001). Cluster-defined HRD patients include stable patients within BRCA1-like and BRCA2-like subtypes. In the right panel, distribution of ovaHRDscar scores across the four categories as in the left panel. Values are displayed for all samples. In each sub-panel the red line represents the threshold of 54 that determines if a sample is classified as HRD or HRP.

We also evaluated the distribution of ovaHRDscar values across the four classification categories (Note Figure 10, right panel). Since ovaHRDscar represents a continuous spectrum with a threshold of 54 used to distinguish HRP from HRD, we aimed to understand how the four groups are positioned relative to this cutoff. Notably, the group classified as HRP by clustering but HRD by ovaHRDscar showed values clustered closer to the threshold. This suggests that ovaHRDscar may tend to overestimate HRD status in some cases, potentially classifying borderline tumors as HRD despite their similar chemotherapy response profiles to HRP patients.

4.5 Transcriptomic analyses

We aimed to explore the six subtypes at the RNA level to identify the biological mechanisms and pathways that characterize each group. First, we matched RNA with DNA samples and

selected only stable patients (general stability). To prevent patients with a larger number of samples from skewing the results, we included up to two samples per patient, prioritizing the highest purity samples from two defined tissue categories: the adnexal compartment (e.g., ovary, fallopian tube) and the metastatic compartment (peritoneum, mesentery, or omentum). This approach was based on the significant differences in transcriptomic profiles between these tissue types and the bias that can occur when only one sample per patient is used.

We analyzed the HRP compartment by comparing each of the three HRP clusters—excluding the non-HGSC cluster—using a one-vs-all approach. Within each subtype, we then examined transcriptomic differences between the adnexal and metastatic compartments. Specifically, we conducted three comparisons:

1. analysis of adnexal tissues to capture subtype-specific features of the originating tumors;
2. analysis of metastatic tissues to assess how subtypes behave at distant sites;
3. comparison between adnexal and metastatic samples within each subtype to explore transcriptomic changes potentially involved in metastatic progression.

Due to the heterogeneity of available tissue sampling, it was not possible to obtain matched samples from the same tissue sites for every patient. As a result, the analysis was performed at the group level rather than through strictly matched patient comparisons.

To identify differentially expressed genes (DEGs) we utilized pydeseq2 (v0.3.4, RRID: SCR_015687) [23] on PRISM (RRID: SCR_027136) epithelial ovarian cancer (EOC) component. Scale factors derived from the PRISM framework [24] were incorporated as size factors and normalized using the geometric mean across samples. We performed pathway activity analysis using DecoupleR (v1.5.0, RRID: SCR_027127) [25], employing the multivariate linear model method decoupler.run_mlm() to infer pathway enrichment scores. For transcription factor activity, we used the CollecTRI gene regulatory network [26], applying the univariate linear model method decoupler.run_ulm() and leveraging the gene-level statistic “stat” from the DESeq2 output.

We also performed over representation analysis (ORA) and a gene set enrichment analysis (GSEA). ORA was conducted on DEGs from decoupleR with a p-value < 0.1 using FDR as multiple correction. GSEA was performed on the Trimmed Mean of M-values (TMM) normalized EOC expression profiles obtained using the conorm (v1.2.0, RRID: SCR_027139) to calculate the functional enrichment scores (GSEApy, v1.1.3, RRID: SCR_025803). We utilized for both the

analyses the hallmark gene sets from the Molecular Signatures Database (MSigDB, RRID: SCR_016863) [27] collection.

4.5.1 Cell types

We used cibersort (RRID: SCR_016955) [28] to evaluate the proportions of different immune cell types in the TME for each bulk RNA sample. We then normalized the proportions using the immune component weights from PRISM [24] and compared the cell types between groups using the Kruskal-Wallis test, with the significance level set at 0.05.

4.5.2 Single cell analyses

The major cell types were identified using KNN graph-based clustering algorithm and canonical cell type markers: epithelial cancer cells (*MUC16*, *PAX8*, *WFDC2*), stromal cells (*COL1A2*, *DCN*, *FGFR1*, *VIM*), and immune cells (*CD14*, *CD3D*, *CD79B*, *CD8A*, *FCER1G*, *HLA-DRA*, *NKG7*, *PTPRC*). The QC thresholds were determined for each cell type separately, and the cells having log2(counts) smaller than the threshold were removed. The threshold was set to 12.5 for epithelial cancer cells, 10 for immune cells, and 11 for stromal cells. Potential doublets were filtered utilizing the DoubletFinder (v2.0.4, RRID: SCR_018771) R package [29]. After ensuring the high quality of the cells, cells were clustered again following standard Seurat workflow.

Cells were assigned into higher resolution of cell types utilizing markers used in [30]. Beside those nine cell types, there was one small cell cluster which consisted of erythroid cells based on marker expression (*HBA1*, *HBA2*, *HBB*). Cell type counts were normalized across all samples. The fibroblast-tumor ratio was calculated by dividing the fibroblast fraction by the cancer cell fraction. For this analysis, only metastatic samples from stable patients were included, as fibroblast proportions differ significantly between primary and metastatic sites. Immune cell analysis, on the other hand, included all samples and was performed by normalizing the abundance of each immune cell type relative to the total immune cell content within each sample.

Proliferation analysis was conducted by first calculating a sample-specific proliferation score, defined as the proportion of cancer cells in S and G2/M phases relative to the total number of cancer cells. Cell cycle phase classification (G1, S, G2/M) was performed using the CellCycleScoring function from Seurat. To avoid bias from patients with multiple samples, only one sample per patient—the one with the highest proliferation score—was included in the

analysis. All statistical analyses were conducted using the Mann-Whitney U-test with FDR correction for multiple comparisons.

4.5.3 Transcriptomics signatures

Proliferation and hypoxia associations were analyzed using signifinder (v1.6.0, RRID: SCR_027141) [31] specifically leveraging the mitotic index score [32], cell cycle signature [33], and hypoxia score [34]. These metrics were applied to the EOC component from bulk RNA-seq data. Comparisons between HRP clusters, using a one-vs-other approach, revealed significantly higher proliferation and hypoxia levels in the Proliferative HRP subtype. Only site-of-origin and metastatic samples from stable patients have been used for the analysis. Results from the cell cycle signatures are shown in Note Figure 11 while the results for the hypoxia in Note Figure 12.

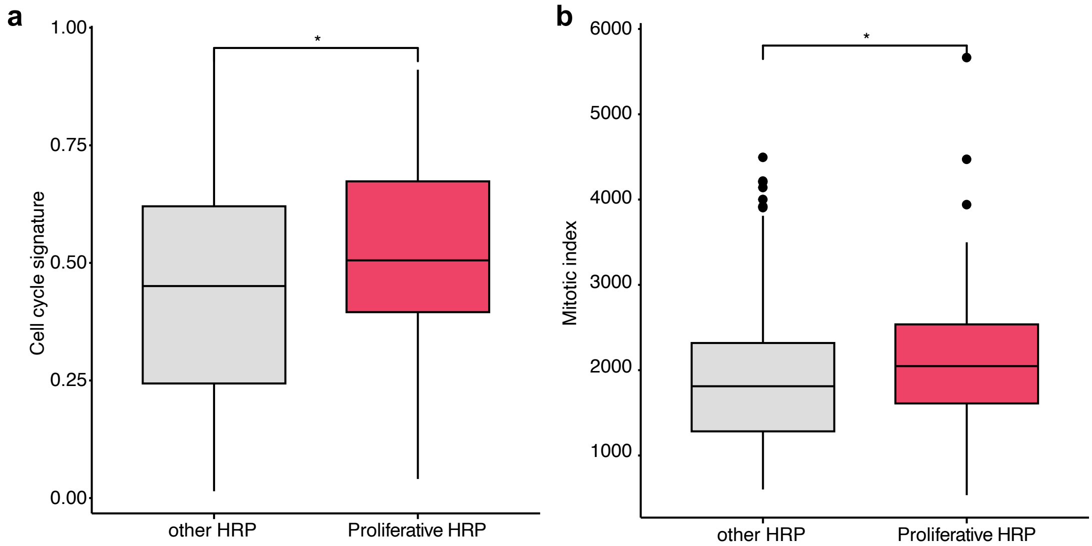

Note Figure 11: cell cycle-associated signature scores in the proliferative HRP cluster patients against the patients in the other two HRP clusters. Comparisons with Wilcoxon test. a, Cell cycle signature from [35] (p = 0.03). b, Mitotic index from [34] (p = 0.03).

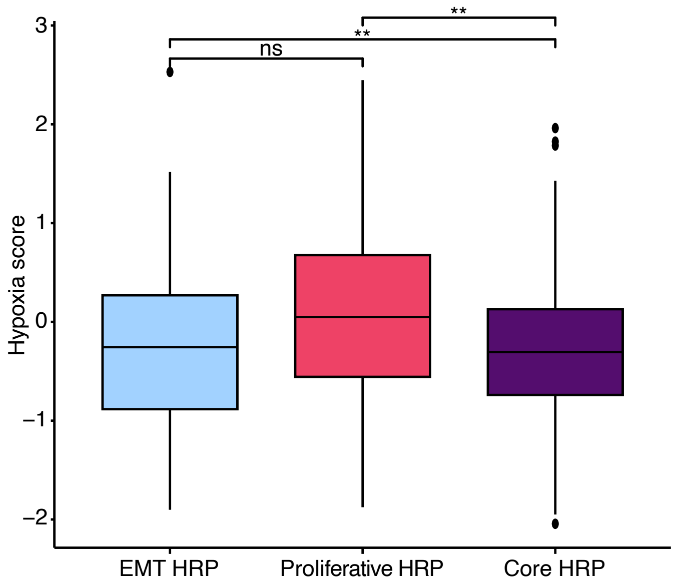

NoteFigure 12: Hypoxic signature score in the HRP clusters. Comparison with pairwise Wilcoxon test with Benjamini-Hochberg correction ($p_{prol\text{-}EMT}$ = 0.005, $p_{prol\text{-}core}$ = 0.005)

4.6 Cell lines characterization for drug sensitivities

4.6.1 Preprocessing

We downloaded WGS sequencing data for HGSC cell lines with wild type (ES2, FUOV1, JHOS2, NIH:OVCAR3, OVCAR4, OVACR8, OV90, SNU119) from SRA (accession number PRJNA523380).

Table7: cell lines identification numbers and common names used.

Cell line ID	Common name	RRID
SRR8639144	ES2	CVCL_AX39
SRR8639235	FUOV1	CVCL_2047
SRR8652066	JHOS2	CVCL_4647
SRR8670698	NIH:OVCAR3	CVCL_0465
SRR8670727	OVCAR4	CVCL_1627
SRR8670745	OV90	CVCL_3768
SRR8670726	OVCAR8	CVCL_1629
SRR8670769	SNU119	CVCL_5014

We realigned the data to GRCh38 without further base quality score recalibration and otherwise processed them as described in the methods section. Somatic mutations and CN calls were obtained with the same tools in tumor-only mode forcing a purity of 1 for PURPLE ploidy/purity estimation.

Somatic variants were filtered to remove possible germline variants using dbSNP (v156, RRID: SCR_002338) [35], gnomAD (v4.1, RRID: SCR_014964) [36] ad COSMIC v100 [13] retaining only those that satisfied at least one of the following conditions:

- Absence from dSNPv156
- In dbSNP and gnomAD v4.1 joint max allele frequency < 0.0001
- In dbSNP and confirmed somatic in COSMIC v100 with 2+ occurrences

Signature attribution was performed as previously described using COSMIC v3.3.1. SBS3 and ID6 were used as HRD indicators and excluded from the analysis OVCAR8, OVCAR4 and SNU119.

4.6.2 Cluster assignment

We built a random forest classifier with scikit-learn (v1.5.2, RRID: SCR_002577) RandomForest using signatures activities from stable patients in HRP clusters. Data was split into a training and a test set keeping all samples from the same patient in the same set with a proportion of

70:30. We performed a randomized search with 10-fold cross-validation to optimize the number of estimators and the maximum tree depth. The best-performing model was identified with a maximum depth of 9 and 96 estimators, achieving a test accuracy of 0.89.

In this regard, cell lines CN and SV calls were used for feature extraction and then signature assignment as described previously. The trained random forest model used the estimated signature activities for clusters assignment obtaining three proliferative HRP cell lines and two core HRP cell lines.

4.6.3 Drug sensitivity analysis

We downloaded cell lines drug screens from DepMap (RRID: SCR_017655) [37] and specifically data regarding the screens Sanger GDSC1 and Sanger GDSC2. For the above-mentioned cell lines, we selected only matching ovarian cancer cells lines and for each study we compared the sensitivities between the clusters. Because IC50 values from the GDSC1 and GDSC2 datasets were not directly comparable, we focused our analysis on GDSC1, which included a broader range of screened compounds. For each drug, we calculated the average half maximal inhibitory concentration (IC_{50}) across tested cell lines and ranked the compounds accordingly. Among the top ten most effective drugs, we identified five histone deacetylase (HDAC) inhibitors and two compounds targeting the G2/M cell cycle checkpoint. To prioritize candidates with potential for both broad efficacy and subtype specificity, we also assessed the variability in IC50 values across cell lines. Given the overlap in functional targets—particularly the role of HDAC inhibitors in modulating the G2/M checkpoint[38,39]—and the need to distinguish responses among HRP subtypes, we decided to further investigate the most downstream effector in this pathway, CHK1.

4.7 Organoids

Patient derived organoids (PDO) were used to estimate drug sensitivities between HGSC subtypes. Long-term organoids were established as previously described [40] and verified to match samples through WGS similarly to tissue samples [41]. Eight organoids (three from the heterogenic HRP cluster, two from the EMT HRP cluster, one from the proliferative HRP cluster and two from BRCA2-like cluster) were used. Testing was performed as described earlier [36], in technical duplicates. Briefly, organoids embedded in Cultrex BME Type 2 were seeded into 384-well Corning Ultra-Low Attachment plates and covered with appropriate growth medium. Medium was replaced every 72-96h. On day 4, CHK1 inhibitor prexasertib was administered in

two concentrations 10 and 50nM for 7 days, with drug replenishment after 4 days of treatment, together with the culture medium change. Cytotoxicities were estimated by fluorescence microscope image analysis using MetaXpress (Molecular Devices) software (RRID: SCR_016654). Survival in each well was estimated by normalization to negative (DMSO, 100% viability) and positive (10uM bortezomib, 0%) controls.

Statistical difference between the heterogenic cluster organoids and other clusters organoids was established using Welch t test with significance level at 0.05.

5 Validation

As an independent validation dataset, we selected 73 patients with 171 samples using the same inclusion criteria applied to the discovery cohort. CIN features were calculated, and signatures were quantified using SigProfilerAssignment, as previously described. Subtype classification was then performed using the random forest model detailed in Section 4.5.2. Next, we compared the distribution of samples across subtypes to that of the discovery cohort and assessed subtype stability at the patient level, following the methodology outlined in Section 4.1.

5.1 Genomic comparison

We validated the genomic composition of the subtypes by examining the distribution of key alterations, including amplifications of *CCNE1*, *AKT2*, and *MYC*; mutations in *CDK12*, *BRCA1*, *BRCA2*, and *RAD51*; as well as the number of genomic breaks and LINE insertions. Consistent with the discovery cohort, *CCNE1* and *MYC* amplifications were distributed across all subtypes, while *AKT2* amplification was largely restricted to the HRP subtypes, with only a single case observed in the HRD group. *BRCA1* mutations were predominantly found in the BRCA1-like HRD subtype, with a few cases also appearing in the BRCA2-like HRD and core HRP subtypes, mirroring the pattern seen in the discovery dataset. In contrast, BRCA2 and RAD51 mutations were exclusive to the BRCA2-like HRD subtype. *CDK12* mutations were similarly subtype-specific, occurring only in the proliferative HRP group. The distribution of genomic breaks closely matched observations from the discovery cohort, with the highest break counts found in the proliferative HRP, BRCA1-like HRD, and BRCA2-like HRD subtypes; intermediate levels in the core HRP subtype; and minimal breaks in the EMT HRP and non-HGSC subtypes.

5.2 Survival analysis

To investigate the prognostic relevance and reproducibility of subtype-specific outcomes, we compared survival differences between the discovery and validation cohorts, focusing on overall survival (OS) and platinum-free interval (PFI). To evaluate potential differences between the two datasets, we first examined their overall impact on survival analysis. We performed a fixed-effect meta-analysis using the R package metafor (v4.8.0, RRID: SCR_003450)[42], comparing Cox regression results from each cohort. The fixed-effect model was chosen given that both datasets were processed and analyzed identically. In both OS and PFI analyses, no significant heterogeneity was detected between cohorts (I^2_{PFI} =1%, Q-test p_{PFI} = 0.6, I^2_{OS} = 0%, Q-test p_{OS} = 0.5; NoteFigure 13), indicating comparable effect sizes across datasets.

Next, we evaluated subtype-specific effects using a random-effect multivariate meta-analysis with the R package mvmeta (v1.0.3, RRID: SCR_027142)[43]. For OS, only the BRCA2-like HRD subtype showed a significantly lower hazard ratio, a result consistently observed in both datasets. No significant heterogeneity was found across subtypes (I^2 =1%, Q-test p = 0.9). For PFI, the EMT HRP subtype was significantly associated with worse outcomes (HR = 2.18, p = 0.004, NoteFigure 14), while other subtypes did not show significant associations. Heterogeneity between datasets remained minimal (I^2 =1%, Q-test p = 0.6), supporting the robustness of the subtype-specific survival patterns across cohorts.

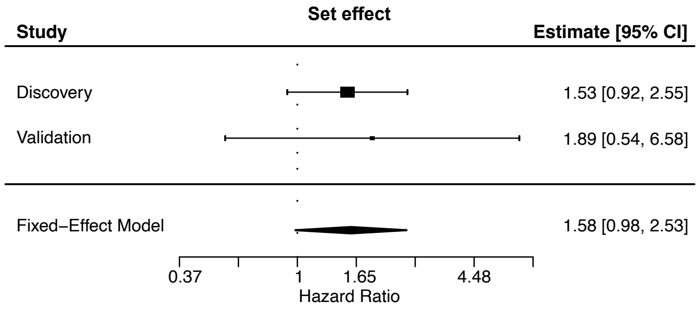

NoteFigure 13: Fixed-effect meta-analysis of subtypes association with overall survival (PFI) across discovery and validation cohorts. Hazard ratios (HRs) and 95% confidence intervals are shown for each dataset, along with the pooled estimated from fixed-effect model.

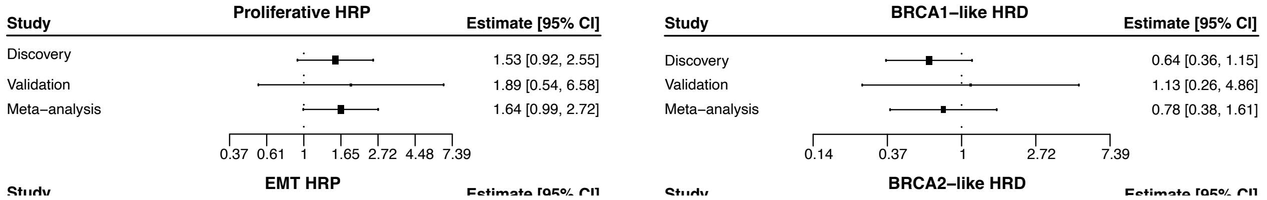

NoteFigure 14: Multivariate meta-analysis of cluster-specific associations with PFI. Encoding as in NoteFigure 13, with pooled estimates derived from a multivariate random-effects meta-analysis. The core HRP cluster was used as the reference group. Hazard ratios were broadly consistent between datasets, with minimal between-study heterogeneity (I^2 = 1.0%, p = 0.6), supporting the reproducibility of cluster- associated survival patterns across independent cohorts. The x-axis is on the hazard ratio scale.

6 References

1. Macintyre, G. *et al.* Copy number signatures and mutational processes in ovarian carcinoma. *Nat Genet* **50**, (2018).
2. Drews, R. M. *et al.* A pan-cancer compendium of chromosomal instability. *Nature* **606**, (2022).
3. Steele, C. D. *et al.* Signatures of copy number alterations in human cancer. *Nature* **606**, (2022).
4. Degasperi, A. *et al.* A practical framework and online tool for mutational signature analyses show intertissue variation and driver dependencies. *Nat Cancer* **1**, (2020).
5. Nik-Zainal, S. *et al.* Landscape of somatic mutations in 560 breast cancer whole-genome sequences. *Nature* **534**, (2016).
6. Shale, C. *et al.* Unscrambling cancer genomes via integrated analysis of structural variation and copy number. *Cell Genomics* **2**, 100112 (2022).
7. Scrucca, L., Fop, M., Murphy, T. B. & Raftery, A. E. mclust 5: Clustering, Classification and Density Estimation Using Gaussian Finite Mixture Models. *R J* **8**, 289 (2016).
8. Cortés-Ciriano, I. *et al.* Comprehensive analysis of chromothripsis in 2,658 human cancers using whole-genome sequencing. *Nat Genet* **52**, (2020).
9. Islam, S. M. A. *et al.* Uncovering novel mutational signatures by de novo extraction with SigProfilerExtractor. *Cell Genomics* **2**, (2022).
10. Cervera, A. *et al.* Anduril 2: upgraded large-scale data integration framework. *Bioinformatics* **35**, 3815–3817 (2019).
11. Van Loo, P. *et al.* Allele-specific copy number analysis of tumors. *Proc Natl Acad Sci U S A* **107**, 16910–16915 (2010).
12. Lavikka, K. *et al.* Deciphering cancer genomes with GenomeSpy: a grammar-based visualization toolkit. *Gigascience* **13**, (2024).
13. Sondka, Z. *et al.* COSMIC: a curated database of somatic variants and clinical data for cancer. *Nucleic Acids Res* **52**, D1210–D1217 (2024).
14. Lahtinen, A. *et al.* Evolutionary states and trajectories characterized by distinct pathways stratify patients with ovarian high grade serous carcinoma. *Cancer Cell* **41**, (2023).
15. Koskela, H. *et al.* HRD related signature 3 predicts clinical outcome in advanced tubo-ovarian high-grade serous carcinoma. *Gynecol Oncol* **180**, 91–98 (2024).
16. Alexandrov, L. B. *et al.* The repertoire of mutational signatures in human cancer. *Nature* **578**, (2020).
17. Mermel, C. H. *et al.* GISTIC2.0 facilitates sensitive and confident localization of the targets of focal somatic copy-number alteration in human cancers. *Genome Biol* **12**, (2011).
18. Gerstung, M. *et al.* The evolutionary history of 2,658 cancers. *Nature 2020 578:7793* **578**, 122–128 (2020).
19. Hadi, K. *et al.* Distinct Classes of Complex Structural Variation Uncovered across Thousands of Cancer Genome Graphs. *Cell* **183**, 197-210.e32 (2020).

20. Allen, R. C. *et al.* An Optimization Framework for Solving Integrated Planning and Scheduling Problems for Dense Energy Carriers. *IFAC-PapersOnLine* **54**, 621–626 (2021).
21. Eisenhauer, E. A. *et al.* New response evaluation criteria in solid tumours: Revised RECIST guideline (version 1.1). doi:10.1016/j.ejca.2008.10.026.
22. Perez-Villatoro, F. *et al.* Optimized detection of homologous recombination deficiency improves the prediction of clinical outcomes in cancer. *NPJ Precis Oncol* **6**, (2022).
23. Muzellec, B., Teleńczuk, M., Cabeli, V. & Andreux, M. PyDESeq2: a python package for bulk RNA-seq differential expression analysis. *Bioinformatics* **39**, (2023).
24. Häkkinen, A. *et al.* PRISM: recovering cell-type-specific expression profiles from individual composite RNA-seq samples. *Bioinformatics* **37**, 2882–2888 (2021).
25. Badia-I-Mompel, P. *et al.* decoupleR: ensemble of computational methods to infer biological activities from omics data. *Bioinformatics advances* **2**, (2022).
26. Müller-Dott, S. *et al.* Expanding the coverage of regulons from high-confidence prior knowledge for accurate estimation of transcription factor activities. *Nucleic Acids Res* **51**, 10934–10949 (2023).
27. Liberzon, A. *et al.* Molecular signatures database (MSigDB) 3.0. *Bioinformatics* **27**, 1739–1740 (2011).
28. Newman, A. M. *et al.* Robust enumeration of cell subsets from tissue expression profiles. *Nature Methods 2015 12:5* **12**, 453–457 (2015).
29. McGinnis, C. S., Murrow, L. M. & Gartner, Z. J. DoubletFinder: Doublet Detection in Single-Cell RNA Sequencing Data Using Artificial Nearest Neighbors. *Cell Syst* **8**, 329-337.e4 (2019).
30. Vázquez-García, I. *et al.* Ovarian cancer mutational processes drive site-specific immune evasion. *Nature* **612**, 778–786 (2022).
31. Pirrotta, S. *et al.* Exploring public cancer gene expression signatures across bulk, single-cell and spatial transcriptomics data with signifinder Bioconductor package. *NAR Genom Bioinform* **6**, (2024).
32. Yang, Z. *et al.* Correlation of an epigenetic mitotic clock with cancer risk. *Genome Biol* **17**, 1–18 (2016).
33. Davoli, T., Uno, H., Wooten, E. C. & Elledge, S. J. Tumor aneuploidy correlates with markers of immune evasion and with reduced response to immunotherapy. *Science* **355**, (2017).
34. Buffa, F. M., Harris, A. L., West, C. M. & Miller, C. J. Large meta-analysis of multiple cancers reveals a common, compact and highly prognostic hypoxia metagene. *Br J Cancer* **102**, 428 (2010).
35. Phan, L. *et al.* The evolution of dbSNP: 25 years of impact in genomic research. *Nucleic Acids Res* **1**, 13–14 (2013).
36. Karczewski, K. J. *et al.* The mutational constraint spectrum quantified from variation in 141,456 humans. *Nature 2020 581:7809* **581**, 434–443 (2020).
37. DepMap 23Q4 Public. https://plus.figshare.com/articles/dataset/DepMap_23Q4_Public/24667905/2.

38. Strait, K. A. *et al.* Histone deacetylase inhibitors induce G2-checkpoint arrest and apoptosis in cisplatinum-resistant ovarian cancer cells associated with overexpression of the Bcl-2-related protein Bad. *Mol Cancer Ther* **4**, 603–611 (2005).
39. Ramaiah, M. J., Tangutur, A. D. & Manyam, R. R. Epigenetic modulation and understanding of HDAC inhibitors in cancer therapy. *Life Sci* **277**, 119504 (2021).
40. Senkowski, W. *et al.* A platform for efficient establishment and drug-response profiling of high-grade serous ovarian cancer organoids. *Dev Cell* **58**, 1106-1121.e7 (2023).
41. The OvaCure Collection - OvaCure. https://www.ovacure.org/en/the-ovacure-collection/.
42. Viechtbauer, W. Conducting Meta-Analyses in R with the metafor Package. *J Stat Softw* **36**, 1–48 (2010).
43. Gasparrini, A., Armstrong, B. & Kenward, M. G. Multivariate meta-analysis for non-linear and other multi-parameter associations. *Stat Med* **31**, 3821–3839 (2012).
