# Supplementary material for: Decoding the Genomic and Functional Landscape of Emerging Subtypes in Ovarian Cancer
Source: Cancer Discov. 2025 Jul 31;15(11):2262–77. doi: 10.1158/2159-8290.CD-25-0652 (PMC12580789; doi:10.1158/2159-8290.CD-25-0652)
Supplement: Supplementary Figures S1-S9 — Supp. Fig. S1 shows signature robustness. Supp. Fig. S2 displays the association of all signatures with homologous recombination and BRCA1/2 mutations. Supp. Fig. S3 depicts the association of signatures with whole genome duplication, amplification of CCNE1, KRAS, MYC, and MECOM and the relationship with survival. Supp. Fig. S4 shows how complex structural variants manifest and in which signatures they are prevalent. Supp. Fig. S5 explains the SCN-G segmentation aberration. Supp. Fig. S6 presents the validation dataset results in terms of survival and genomic characteristics. Supp. Fig. S7 displays transcriptomics analysis results for the proliferative HRP subtype. Supp. Fig. S8 depicts the transcriptomic results for the EMTHRP subtype. Supp. Fig. S9 contains the results from the transcriptomic analysis for the core HRP subtype. [file cd-25-0652_supplementary_figures_s1-s9_suppsf1.pdf]

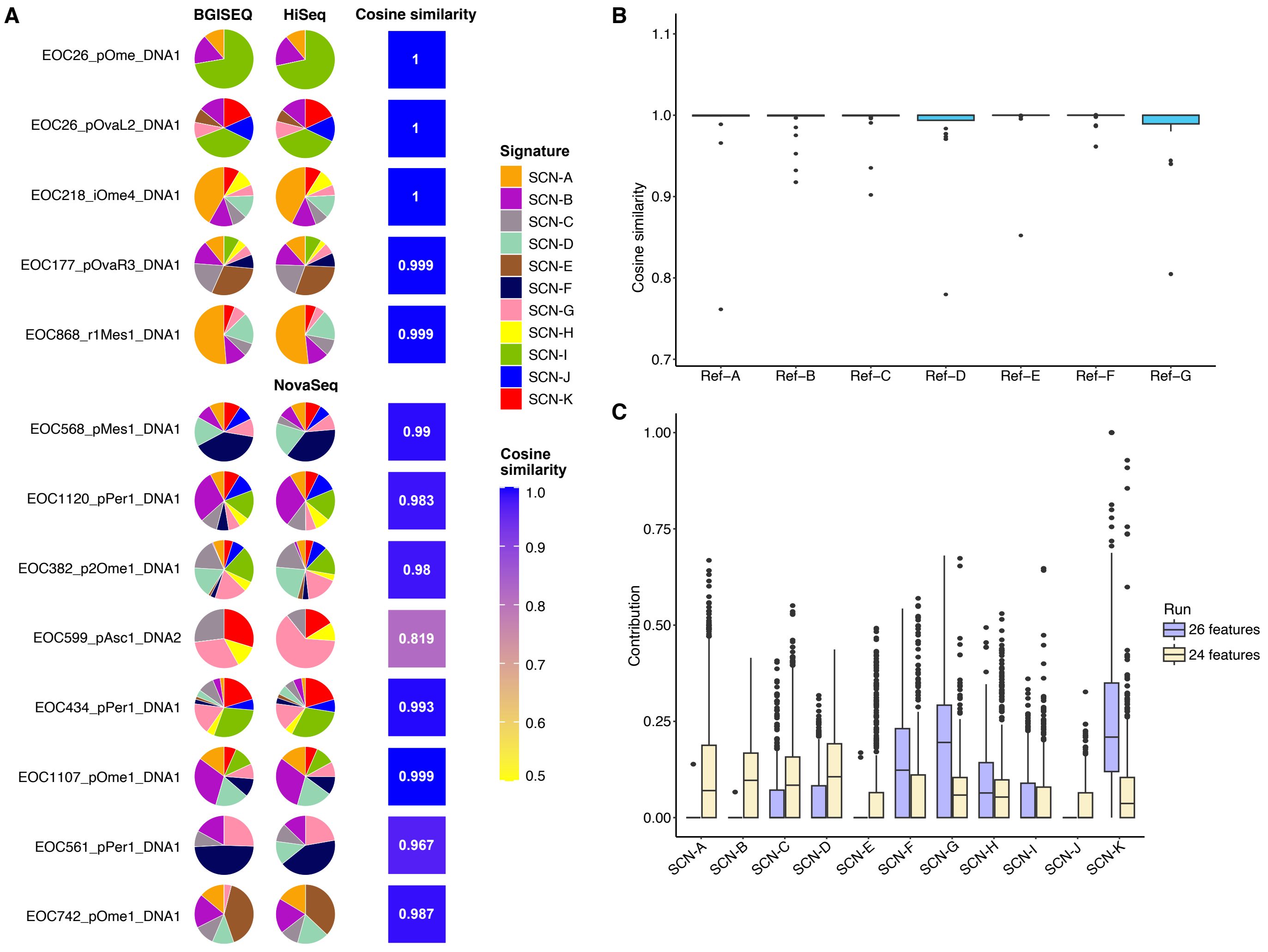

Supplementary Fig. S1 | Signatures robustness. **A**, Robustness across different sequencing platforms. Signature activities of samples from three sequencing platforms are shown as pie plots (left) and their cosine similarity as a heatmap (right). **B**, Cosine similarity between each run and its reference run across groups. To evaluate the robustness of signature extraction, SigProfilerExtractor parameters were varied using a subset of 50 samples. Runs were grouped based on reference signatures obtained with default parameters. **C**, Comparison of signature activity distributions with and without structural variation features. SV-related features were removed (26 remaining) and recalculated using GATK-ASCAT segmentation, with the results compared to the original set derived from 44 features and GRIDSS-PURPLE segmentation.

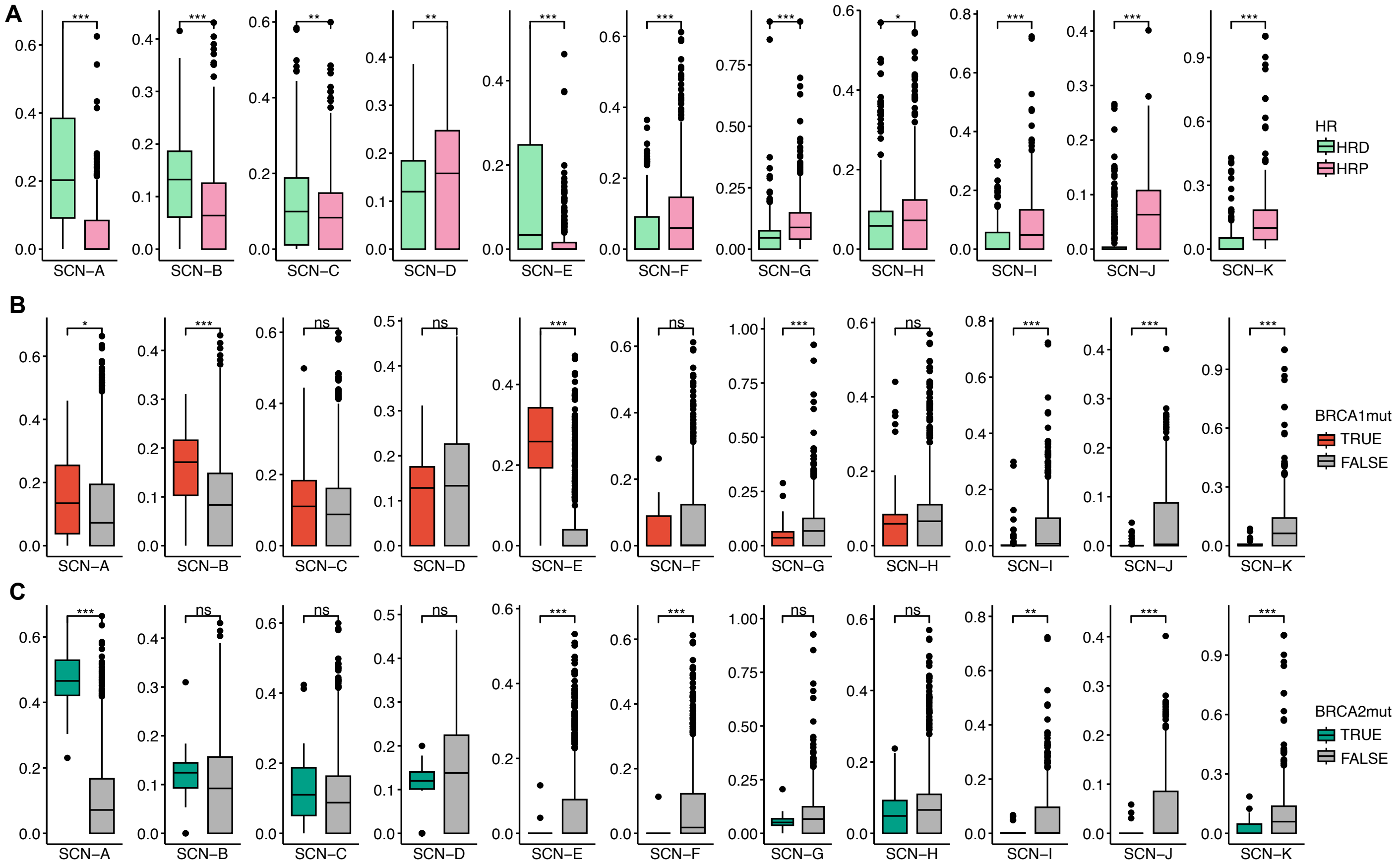

Supplementary Fig. S2 | Complete homologous recombination and *BRCA1/2* associations. **A**, Comparison of signature activity between homologous recombination deficient (HRD) and proficient (HRP) samples. The HR status of each patient is defined based on the activity of the SBS3 mutational signature, as explained in the Supplementary Notes. Significance was calculated using Mann-Whitney U-tests with FDR correction. **B**, Signature activities in *BRCA1*-deficient and *BRCA1*-normal patients. Significance test and levels as in A. **C**, Signatures activities in *BRCA2*-mutated and *BRCA2*-normal patients. Significance test and levels as in A.

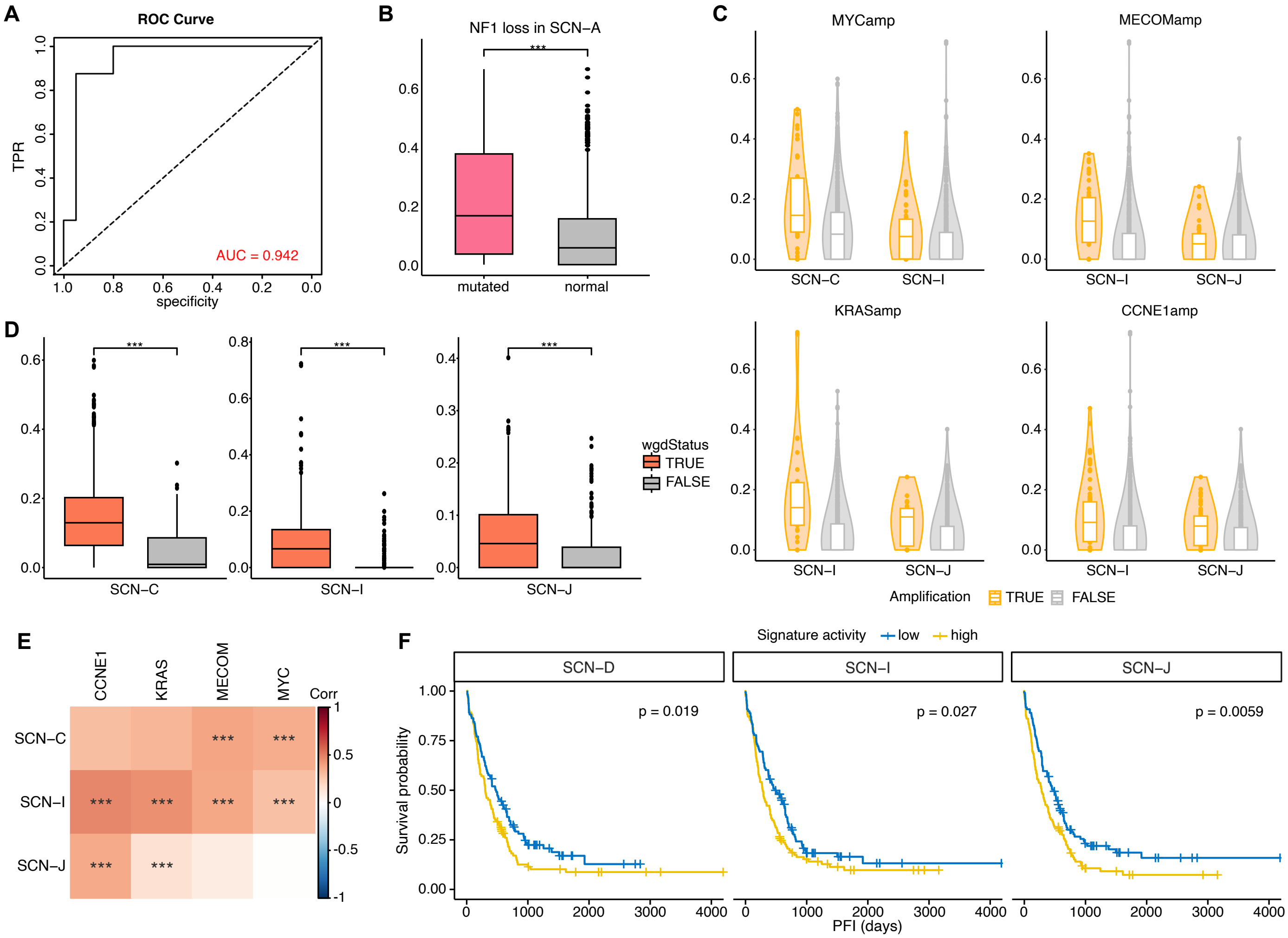

Supplementary Fig. S3 | Biological association of SCN signatures. **A**, ROC curve showing the performance of a logistic regression model using SCN-A, SCN-B, and SCN-E signatures to classify homologous recombination status based on the SBS3 mutational signature. The plot illustrates the true positive rate vs. specificity and includes the AUC value as a measure of overall model performance. **B**, SCN-A signature activity in patients bearing *NF1* mutation or deletion and *NF1*-normal patients. Mann-Whitney U-test with FDR correction (p = 1.19e-14). **C**, Signatures activities in samples with amplified and normal state *MYC*, *MECOM*, *KRAS*, and *CCNE1*. Significance levels were calculated using Mann-Whitney U-tests with FDR correction. *MYC* SCN-C (p = 7.3e-5) and SCN-I (p = 0.001), *MECOM* SCN-I (p = 4.5e-7) and SCN-J (p =0.028), *KRAS* SCN-I (p = 7e-8) and SCN-J (p =3.9e-4) and *CCNE1* SCN-I (p = 4.4e-10) and SCN-J (p = 1.1e-8). **D**, Enrichment of signatures activities SCN-C (p = 1.88e-30), SCN-I (p = 2.83e-34), and SCN-L (p = 9.12e-12) in whole-genome duplicated samples. Statistical tests as in C. **E**, Correlation plot between signatures SCN-I, SCN-J, and SCN-C with frequently focal amplified genes. Encoding as in Fig. 2A. **F**, Kaplan Meier survival analysis of HRP-relevant signatures with log-rank p-values. Encoding and calculation as in Fig. 2C.

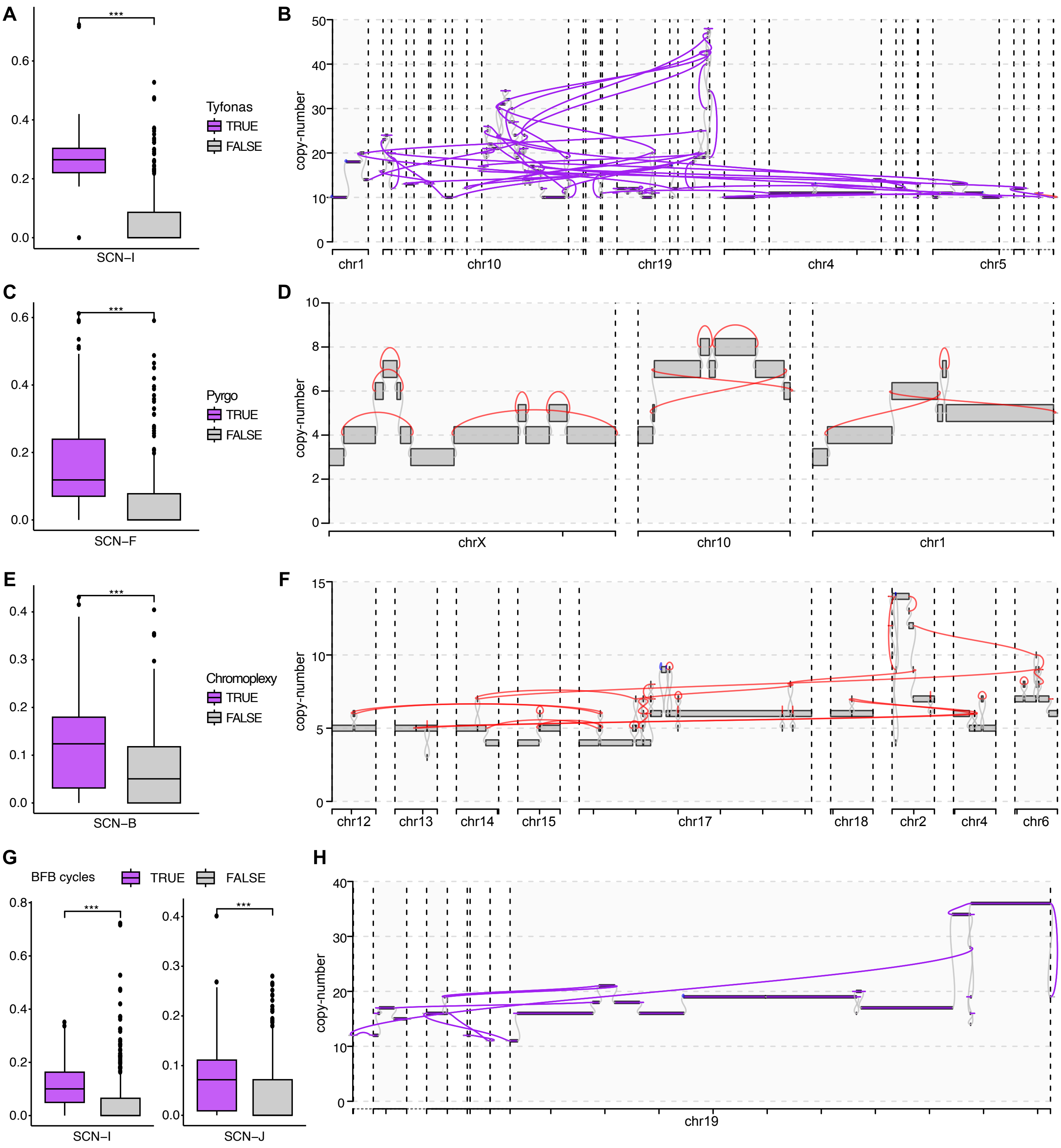

A
B
C
D
E
F
G
H
Tyfonas
TRUE
FALSE
Pyrgo
TRUE
FALSE
Chromoplexy
TRUE
FALSE
BFB cycles
TRUE
FALSE
***
SCN–I
SCN–F
SCN–B
SCN–J
copy-number
chr1
chr10
chr19
chr4
chr5
chrX
chr12
chr13
chr14
chr15
chr17
chr18
chr2
chr6

Supplementary Fig. S4 | Complex SVs prevalence and aspect. **A**, Tyfonas prevalence in patients with high activities of SCN-I. Tyfonas are “typhoons” of fold-back inversions, encoded as a discrete variable (presence or absence). Mann-Whitney U test (p = 4.92e-3). **B**, Tyfona event representation in patient EOC888 spanning multiple chromosomes, with the center in chr10. Purple lines represent the connections between segments, and vertical dashed lines set the specific regions of the chromosomes in which the segments are located. **C**, Pyrgo, which are towers of amplifications, are prevalent in patients with high activities of SCN-F (p = 2.01e-29). Encoding and statistical test as is A. **D**, Representation of pyrgo three events in patient EOC130. Red lines represent the links between the breakpoints. Dashed lines have the same function as in B. **E**, Activities of SCN-B signature in samples with and without chromoplexy events. Significant test as in A (p = 2.92e-14). **F**, Representation of a chromoplexy event in patient EOC292. Plot elements like in B. **G**, SCN-I (p = 4.99e-25) and SCN-J (p = 4.44e-16) signature activities in samples with and without breakage-fusion-bridge (BFB) cycles. Encoding and statistical test as in A **H**, Representation of a BFB cycle event in patient EOC890 in chromosome 19. Encoding as in B.

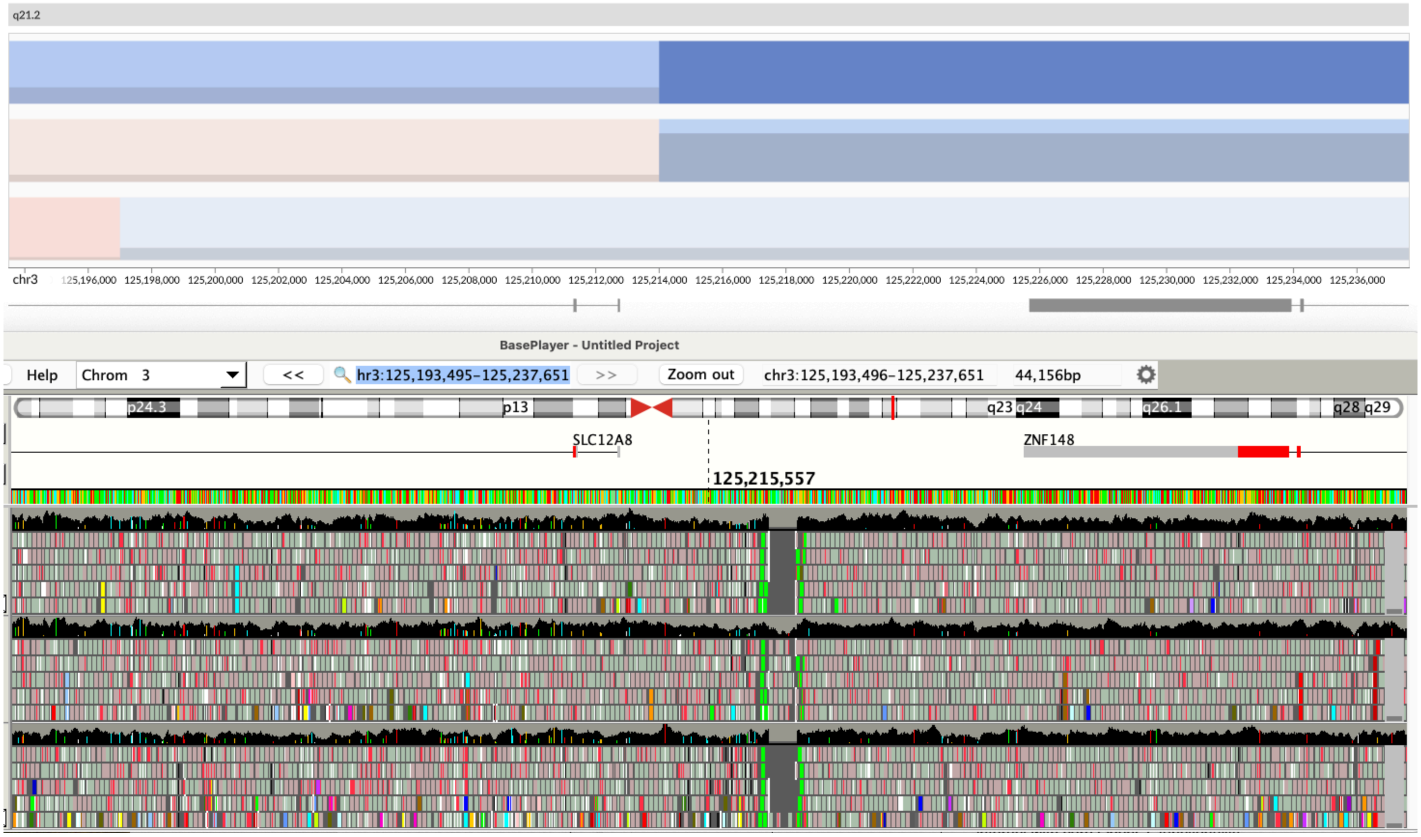

Supplementary Fig. S5 | Segmentation in SCN-G. SCN-G identifies samples in which PURPLE fails to build a reliable segmentation model. In the upper part, a GenomeSpy visualization extract of chromosome 3 of samples EOC1099_pAsc2_DNA3, EOC871_r1Asc1_DNA1, and EOC733_iAsc1_DNA1. In the bottom part, the same samples are visualized in the same genomic regions with BasePlayer, a tool for visualizing genomic data. Each sample track is composed of a coverage track and the aligned reads at each position. The deletions highlighted by GenomeSpy in the three samples start close to a small deletion visible in BasePlayer, marked by the green reads (discordant read pairs) and low coverage. However, in the adjacent region, there is no evidence of a long deletion in the coverage tracks or in the reads, as predicted in the segmentation.

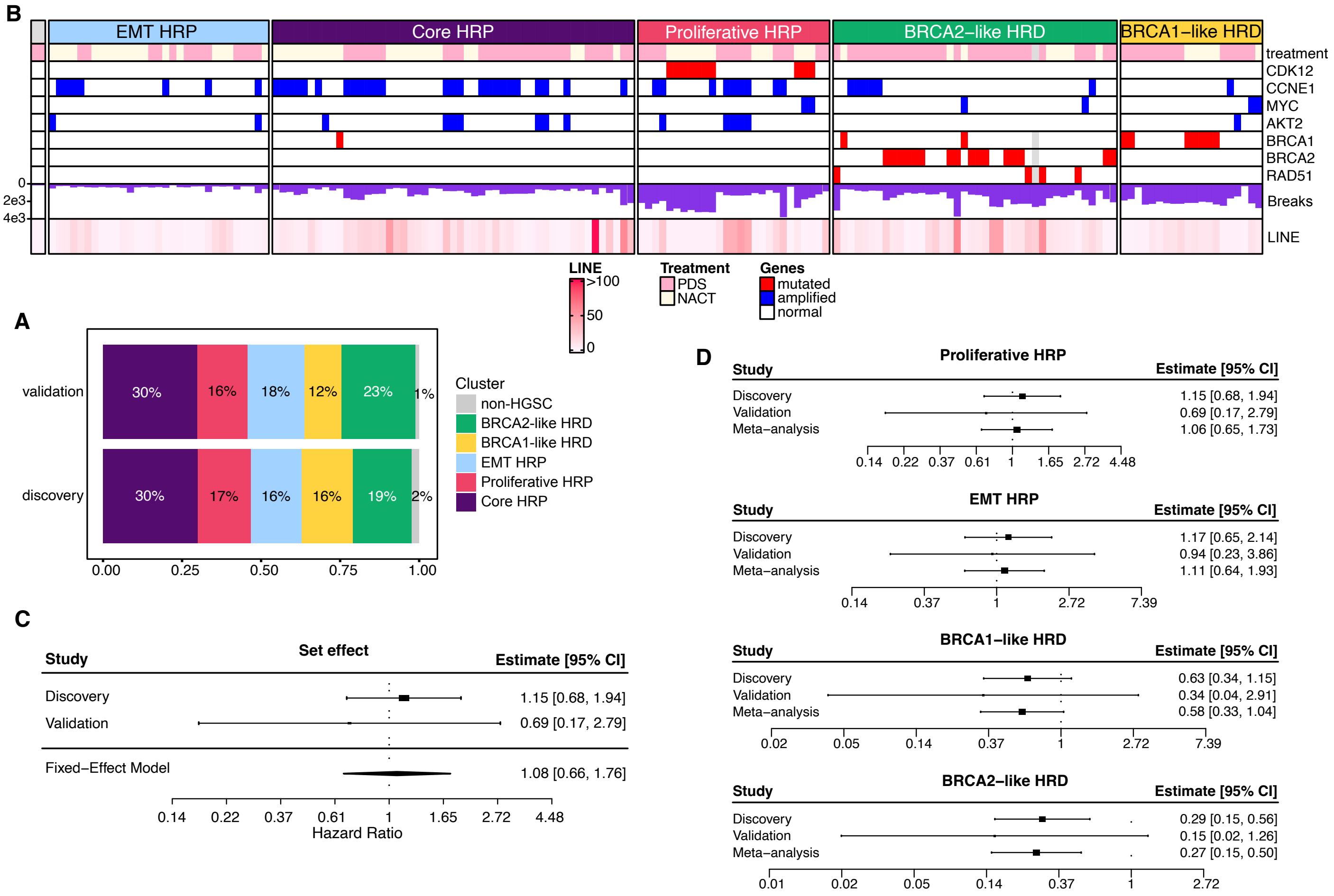

Supplementary Fig. S6 | Validation cohort. A, Composition of the discovery and validation cohorts. The bars and the percentages show the number of samples in each subtype per cohort. The composition of the two cohorts is very similar, with the highest difference observed between BRCA1-like and BRCA2-like HRD subtypes. **B**, Genomic landscape of validation samples, clustered into the subtypes. PDS patients were prevalent in BRCA2-like and BRCA1-like HRD subtypes as in the discovery cohort. Genomic alterations also reflected the findings in the discovery cohort with *CDK12*mut in the proliferative HRP subtype, *BRCA2*mut, and *RAD51*mut in the BRCA2-like HRD subtype, and *AKT2*amp mostly in the HRP subtypes. Higher amounts of breaks were observed in the proliferative and HRD subtypes, while the lowest amounts were in non-HGSC and EMT HRP subtypes. **C**, Fixed-effect meta-analysis of subtype association with overall survival (OS) across discovery and validation cohorts. Hazard ratios (HRs) and 95% confidence intervals are shown for each dataset, along with the pooled estimate from the fixed-effect model. Heterogeneity was low ($Q(1) = 0.44$, $p = 0.59$) with $I^2 = 0\%$, supporting the use of a fixed-effect model. **D**, Multivariate meta-analysis of cluster-specific associations with OS. Encoding as in panel c, with pooled estimates derived from a multivariate random-effects meta-analysis. The core HRP cluster was used as the reference group. Hazard ratios were broadly consistent between

datasets, with minimal between-study heterogeneity ($I^2 = 1.0\%$, $p = 0.95$), supporting the reproducibility of cluster-associated survival patterns across independent cohorts. The x-axis is on the hazard ratio scale.

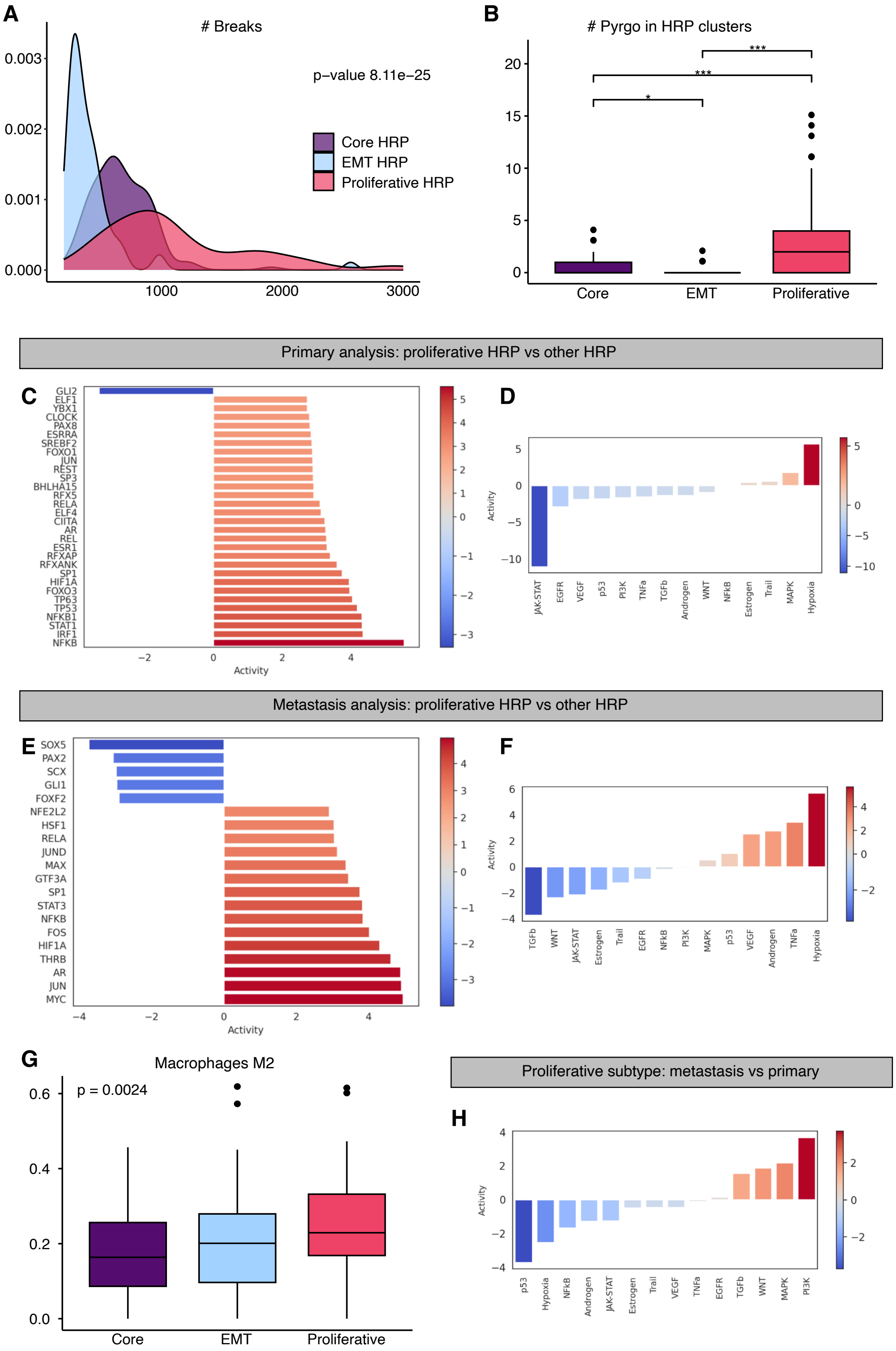
A
Breaks
p–value 8.11e–25
Core HRP
EMT HRP
Proliferative HRP
0.003
0.002
0.001
0.000
1000
2000
3000
B
Pyrgo in HRP clusters
***
***
*
20
15
10
5
0
Core
EMT
Proliferative
Primary analysis: proliferative HRP vs other HRP
C
GLI2
ELF1
YBX1
CLOCK
PAX8
ESRRA
SREBF2
FOXO1
JUN
REST
SP3
BHLHA15
RFX5
RELA
ELF4
CIITA
AR
REL
ESR1
RFXAP
RFXANK
SP1
HIF1A
FOXO3
TP63
TP53
NFKB1
STAT1
IRF1
NFKB
Activity
D
Activity
JAK-STAT
EGFR
VEGF
p53
PI3K
TNFa
TGFb
Androgen
WNT
NFkB
Estrogen
Trail
MAPK
Hypoxia
Metastasis analysis: proliferative HRP vs other HRP
E
SOX5
PAX2
SCX
GLI1
FOXF2
NFE2L2
HSF1
RELA
JUND
MAX
GTF3A
SP1
STAT3
NFKB
FOS
HIF1A
THRB
AR
JUN
MYC
Activity
F
Activity
TGFb
WNT
JAK-STAT
Estrogen
Trail
EGFR
NFkB
PI3K
MAPK
p53
VEGF
Androgen
TNFa
Hypoxia
G
Macrophages M2
p = 0.0024
0.6
0.4
0.2
0.0
Core
EMT
Proliferative
Proliferative subtype: metastasis vs primary
H
Activity
p53
Hypoxia
NFkB
Androgen
JAK-STAT
Estrogen
Trail
VEGF
TNFa
EGFR
TGFb
WNT
MAPK
PI3K

Supplementary Fig. S7 | Transcriptomic analysis in the proliferative subtype. **A**, Number of breaks per sample in the HRP subtypes. Unstable samples have been excluded. Statistical significance was assessed with Kruskal-Wallis test. **B**, Differences in the number of pyrgos between HRP subtype samples. Patient selection as in A. Statistical significance was evaluated with pairwise Wilcoxon tests. **C**, **D**, Transcription factor (CollecTRI) and pathway (progeny) activities respectively from the same comparison as in a. Interestingly, high hypoxia and low JAK-STAT pathways and upregulation of AR and NFKB in the transcription factors. **E**, **F**, Same encoding as in C, D, but referred to the comparison between the proliferative subtype versus the other HRP subtypes in the metastatic tissues. Notably, there is a strong upregulation of the hypoxia and androgen signaling pathway in this compartment, while the estrogen pathway is downregulated. This is also supported by the high activity of AR and HIF1A in the transcription factor analysis. **G**, Enrichment of macrophages M2 in the proliferative HRP subtype. Statistical test and patient selection as in A. **H**, Pathway analysis in the proliferative subtype, comparing primary versus metastatic tissues. The hypoxia pathway is strongly upregulated in the metastatic tissues.

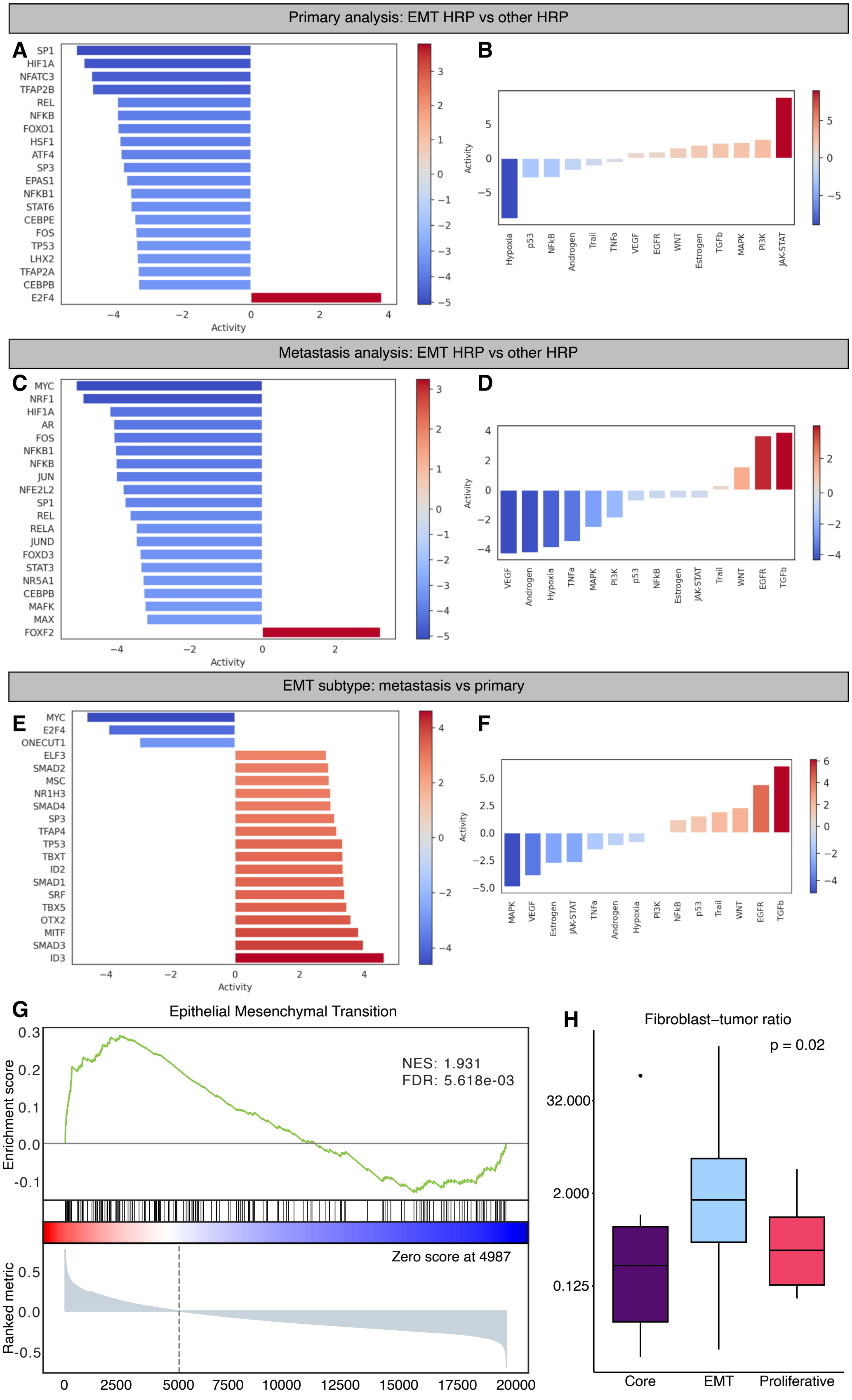
Primary analysis: EMT HRP vs other HRP
A
SP1
HIF1A
NFATC3
TFAP2B
REL
NFKB
FOXO1
HSF1
ATF4
SP3
EPAS1
NFKB1
STAT6
CEBPE
FOS
TP53
LHX2
TFAP2A
CEBPB
E2F4
Activity
B
Activity
Hypoxia
p53
NFkB
Androgen
Trail
TNFa
VEGF
EGFR
WNT
Estrogen
TGFb
MAPK
PI3K
JAK-STAT
Metastasis analysis: EMT HRP vs other HRP
C
MYC
NRF1
HIF1A
AR
FOS
NFKB1
NFKB
JUN
NFE2L2
SP1
REL
RELA
JUND
FOXD3
STAT3
NR5A1
CEBPB
MAFK
MAX
FOXF2
Activity
D
Activity
VEGF
Androgen
Hypoxia
TNFa
MAPK
PI3K
p53
NFkB
Estrogen
JAK-STAT
Trail
WNT
EGFR
TGFb
EMT subtype: metastasis vs primary
E
MYC
E2F4
ONECUT1
ELF3
SMAD2
MSC
NR1H3
SMAD4
SP3
TFAP4
TP53
TBXT
ID2
SMAD1
SRF
TBX5
OTX2
MITF
SMAD3
ID3
Activity
F
Activity
MAPK
VEGF
Estrogen
JAK-STAT
TNFa
Androgen
Hypoxia
PI3K
NFkB
p53
Trail
WNT
EGFR
TGFb
G
Epithelial Mesenchymal Transition
Enrichment score
NES: 1.931
FDR: 5.618e-03
Zero score at 4987
Ranked metric
0
2500
5000
7500
10000
12500
15000
17500
20000
H
Fibroblast−tumor ratio
p = 0.02
32.000
2.000
0.125
Core
EMT
Proliferative

Supplementary Fig. S8 | Transcriptomic analysis in the EMT subtype. **A**, **B,** Transcription factor (CollecTRI) and pathway (progeny) activities, respectively, from the comparison between EMT subtype versus other HRP subtypes in the primary tissues. Importantly, there is a significant downregulation of transcription factor targets, including HIF1A and EPAS1, which is supported by the low activity of the hypoxia pathway. **C**, **D**, Same encoding as A, B, but referred to the comparison between the EMT subtype versus the other HRP subtypes in the metastatic tissues. Similarly to the primary tissues, there is a strong downregulation of transcription factor targets. Notably, TGFb and EGFR pathways are upregulated. **E**, **F**, Same encoding as A, B, but referred to the comparison between the primary tissues versus the metastatic tissues of the EMT subtype. Similarly to the metastatic tissues analysis, there is strong activation of TGFb pathway. **G**, GSEA revealing significant enrichment of the epithelial-to-mesenchymal transition pathway. The positive normalized enrichment score (NES) indicates higher activity in metastatic samples. Two-sided p-value with Benjamini-Hochberg correction for multiple testing. **H**, Fibroblast-tumor ratio of the HRP subtypes (log-scale). Unstable samples have been excluded. Statistical significance was assessed with Kruskal-Wallis test.

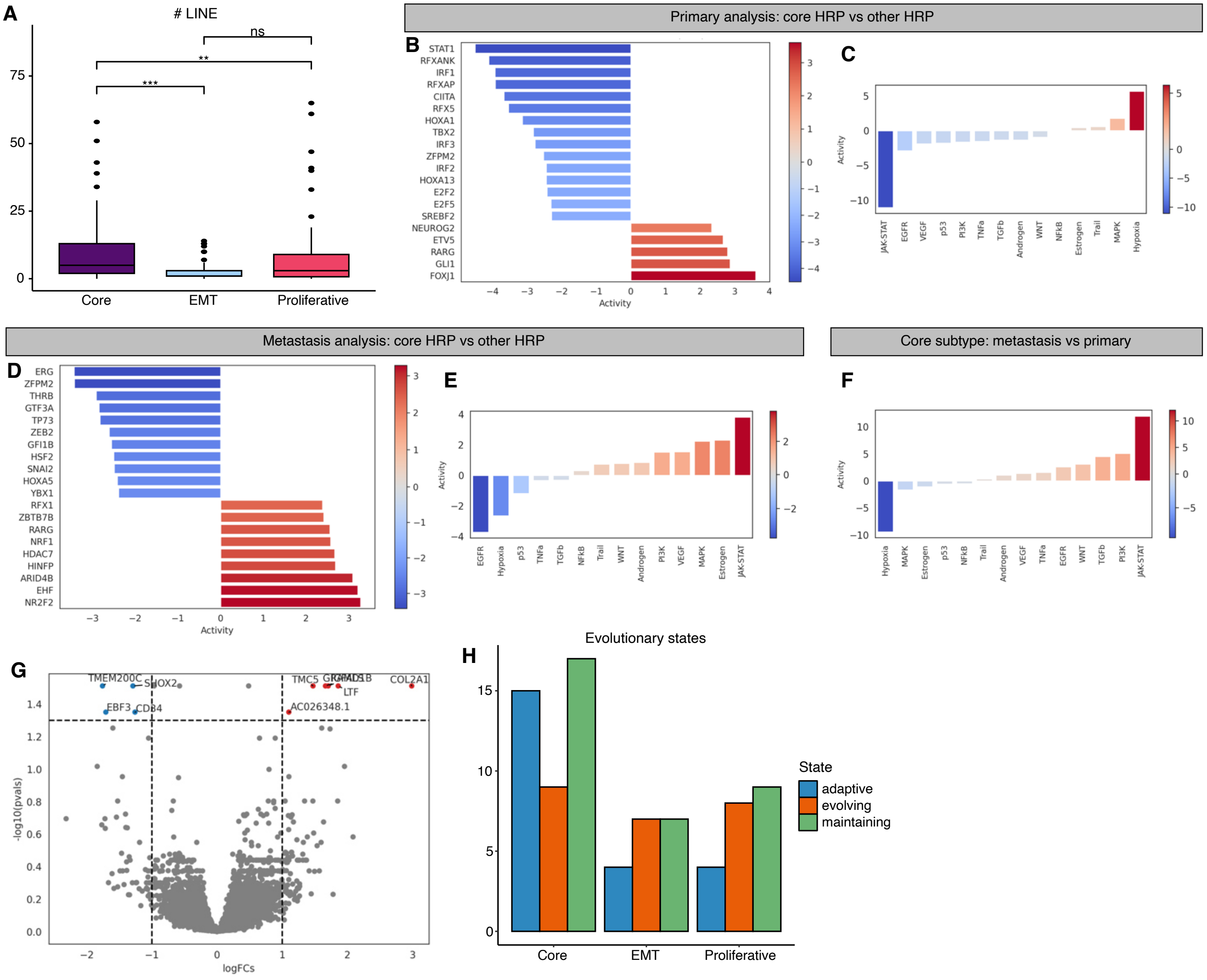

Supplementary Fig. S9 | Transcriptomic analysis in the core subtype. **A**, Differences in the number of LINE events in samples from the HRP subtypes. Statistical significance was evaluated with pairwise Wilcoxon tests. **B**, **C**, Transcription factor (CollecTRI) and pathway (progeny) activities, respectively, derived from the comparison of core subtype versus other HRP subtypes in the primary tissues. Notably, in the transcription factor activities, RFX complex is strongly downregulated. Moreover, pathway analysis reveals upregulation of hypoxia and downregulation of JAK-STAT pathway. **D**, **E**, Same encoding as B, C, but referred to the comparison between the core subtype versus the other HRP subtypes in the metastatic tissues. Interestingly, the hypoxia and JAK-STAT pathways exhibit inverted directions compared to the primary tissue comparison with the other HRP subtypes. **F**, Pathway analysis referred to the comparison between metastatic and primary tissue in the core HRP subtype. JAK-STAT activity is increased in the metastatic compartment, while in the primary tissues there is upregulation of the hypoxia pathway. **G**,

Volcano plot from the comparison of core subtype versus other HRP subtypes in the metastatic tissues differential expression analysis (DEA). Interestingly, only a few genes are differentially expressed, highlighting the heterogeneity of the core HRP metastatic tissues compared to the other subtypes. **H**, Barplot showing the counts of the evolutionary states (3) in the HRP groups, displaying a prevalence of adaptive patients in the core subtype.
